# Discrepancy in scientific authority and media visibility of climate change scientists and contrarians

**DOI:** 10.1038/s41467-019-09959-4

**Published:** 2019-08-13

**Authors:** Alexander Michael Petersen, Emmanuel M. Vincent, Anthony LeRoy Westerling

**Affiliations:** 1 Management of Complex Systems Department, Ernest and Julio Gallo Management Program, School of Engineering, University of California, Merced, CA 95343 USA; 2 Medialab, Sciences Po, Paris, 75007 France; 3 Center for Climate Communication, University of California, Merced, CA 95343 USA; 4 Sierra Nevada Research Institute, University of California, Merced, CA 95343 USA

**Keywords:** Computational science, History, Climate change

## Abstract

We juxtapose 386 prominent contrarians with 386 expert scientists by tracking their digital footprints across ∼200,000 research publications and ∼100,000 English-language digital and print media articles on climate change. Projecting these individuals across the same backdrop facilitates quantifying disparities in media visibility and scientific authority, and identifying organization patterns within their association networks. Here we show via direct comparison that contrarians are featured in 49% more media articles than scientists. Yet when comparing visibility in mainstream media sources only, we observe just a 1% excess visibility, which objectively demonstrates the crowding out of professional mainstream sources by the proliferation of new media sources, many of which contribute to the production and consumption of climate change disinformation at scale. These results demonstrate why climate scientists should increasingly exert their authority in scientific and public discourse, and why professional journalists and editors should adjust the disproportionate attention given to contrarians.

## Abstract (web summary)

The role of climate change (CC) contrarians is neglected in climate change communication studies. Here the authors used a data-driven approach to identify CC contrarians and CC scientists and found that CC scientists have much higher citation impact than those for contrarians but lower media visibility.

## Introduction

Since the early 2000s there has been little disagreement among scientific experts over the fundamental evidence supporting the existence, origin, and societal significance of anthropogenic climate change (CC)^1–4^. Yet, while an anthropogenic cause is supported by an overwhelming majority of climate change scientists (CCS)^5^, climate change contrarians (CCC) have successfully organized a strong voice within politics and science communication in the United States^6,7^.

Historians of science have detailed the political origins of the CCC movement, documenting how its strategic efforts succeeded in distorting the science-based narrative on multiple fronts, e.g., by promoting the idea that there is a lack of scientific consensus concerning anthropogenic CC^6,8–12^, despite the fact that objective research has found little evidence for such a claim. One study comparing consensus scientists with unconvinced scientists found that the 2–3% of researchers unconvinced by evidence for anthropogenic CC were not only small in group size but also had substantially lower levels of authority in the CC literature^10^. Another study surveying ∼3000 earth scientists found the highest levels of CC consensus to be among the most expert climatologists^5^.

Public confusion over science affects various other domains^13^, in addition to CC communication^14^, and requires a better understanding of the human, social, and technological factors that facilitate widespread disinformation efforts^15–18^. One salient human factor that contributes to the public’s susceptibility to information manipulation is cognitive bias. A particularly relevant example is motivated reasoning—the tendency for individuals to bias their judgements according to personal- and group-level values, even when faced with documented facts^19–21^. Another class of factors are prominent external influences, owing to elite political cues^22^, ideological biases^23,24^, cultural worldviews^25^, and even personal weather experiences^26,27^. Not least among these external factors is the news media^15^, which has a longstanding and dominant role empowering cultural politics^28^. A third decisive technological factor is the paradigm of new media and the nearly boundless scalability of content distribution across the internet. Even in the case where individuals have complete control in choosing their sources of information, they are nevertheless susceptible to significant disparities in content production in addition to being susceptible to media coverage that is disproportionate to the authority and number of scientists holding the consensus viewpoint. Recent research highlights the ramifications of this problem, finding that the acceptance of CC increases (respectively decreases) with consumption of media content that acknowledges (respectively dismisses) CC realities, other factors being equal^24^. Susceptibility to information manipulation may continue to be a serious problem until society fully adapts to managing the sheer range and volume of new media sources. As such, addressing the opportunities and threats facing CC communication requires an integrated understanding of these human, social, and technological factors.

Accordingly, the literature on CC communication is multi-disciplinary. Research efforts draw on a wide range of methods that typically target a single entry point—such as applying content and meta-analysis methods to select collections of scientific publications^2,3,10,29^, news media articles^7–9,12,28,30–34^, or surveys^4,22,23^ or by developing behavioral experiments and survey instruments^5,11,19,24,25,35^. For example, applying in-depth content analysis to select media article sets, researchers identified common factors among skeptical critics, estimated the percentage of CC articles that contain skeptical elements, and developed a typology of CC skeptics^30^. Building on this framework, another recent study reports that contrarians have strategically shifted away from their external narrative—initially based upon challenging fundamental tenets of CC science (e.g., its anthropogenic origins), thereby positioning themselves as skeptics with legitimate scientific motives for dissent—to instead challenging assessments of CC impacts in an effort to impede the development of proactive regulations^33^. However, a separate large-scale analysis of internal documents from 19 contrarian organizations shows that the inward contrarian narrative is still rather focused on CC science, with the relative frequency of science-related topics increasing relative to policy-related topics over the period 2009–2013^34^.

We complement these extensive efforts by investigating the degree to which socio-technical factors facilitate the visibility and emergence of authority among contrarian claims-makers^36^. To address this literature gap, we focus our analysis on a group of 386 prominent contrarians, denoted both individually and collectively by CCC. We compare these CCC with 386 prominent scientists active in CC research, denoted hereafter by CCS. These experts in CC science serve as an objective measurement baseline for juxtaposing visibility in the media with authority in the scientific domain. To operationalize this integrative comparison, we collected two large datasets through 2016, comprised of ∼200,000 CC research articles from the Web of Science (WOS) and ∼100,000 English-language CC media articles from the Media Cloud (MC) project^37^. By focusing on a fixed set of individuals, we leverage large-scale data-driven methods of computational social science^38^ in an effort to reveal individual-, pair-wise-, and group-level phenomena at the intersection of science and the media.

In what follows, we characterize and compare these CC actors at various levels of aggregation: first, by comparing their scientific authority and media visibility at both the individual and group levels; and second, by mapping their associations that are manifest in media co-visibility networks and scientific co-citation networks. Our approach accounts for the variation in visibility across a wide range of sources, from main-stream to non-mainstream sources. By simultaneously accounting for each individual’s scientific authority, our quantitative analysis contributes to the CC communication literature by revealing the degree to which prominent contrarian voices benefit from the scalability of new media, in particular the large number of second-tier news sources and blogs that do not implement rigorous information quality assessment standards. Such disproportionate media visibility of contrarian arguments and actors not only misrepresents the distribution of expert-based beliefs^28,36,39^, it also manifestly undermines the credible authority of career CCS experts and reinforces the trend of CCC presiding over public scientific discourse^40^, which all together hinders prospects for rapid public action on CC^41^.

## Results

### Dataset construction focusing around individuals

**Fig. 1:**
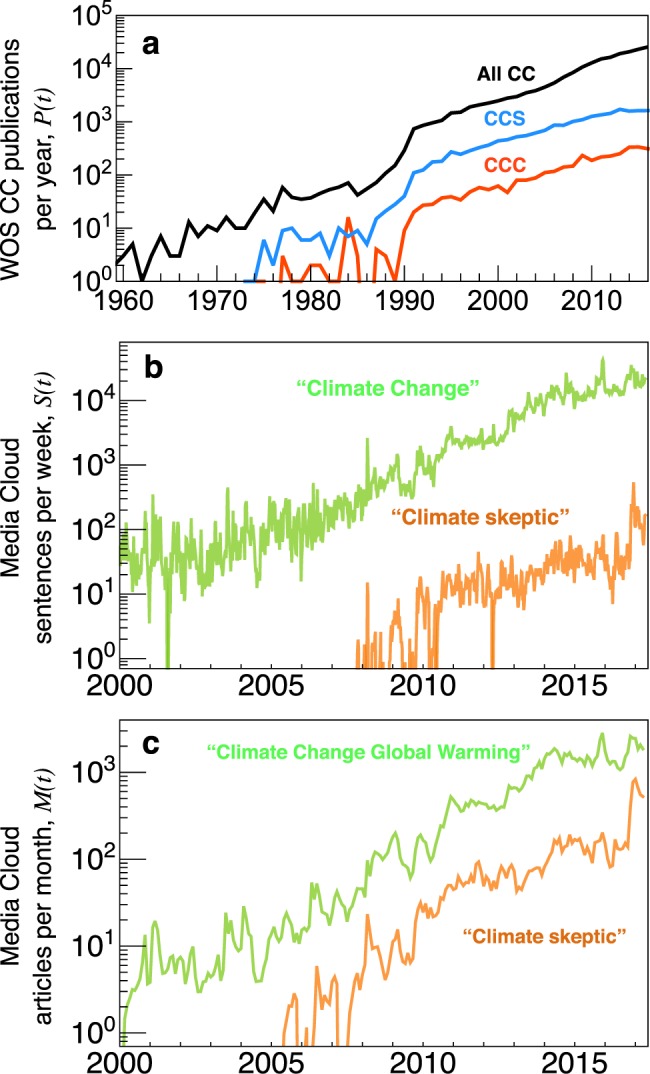
Growth of research output and media production relating to climate change (CC). **a** Number *P*(*t*) of CC publication indexed by Web of Science by year *t*, with 93% published after 2000. The approximate linearity on linear-log axes indicates exponential growth with notable upticks in the late 1980s and late 2000s. **b** The prominence of three CC-related terms, measured by the number of sentences (per week) across all media articles indexed by Media Cloud. (**c**) Total number of media articles indexed by Media Cloud (per month, across all media sources). Exponential smoothing is used in **b** and **c** to tame the noise at the week and month resolution

CC research and media coverage have grown steadily over the last quarter century, drawing on a range of actors from the scientific, public, media, and policy domains^36^. According to MC project^37^ data reported in Fig. 1, the term “climate change” is currently used in approximately 10^4^ media article sentences per week, roughly 100 times as much as the term “climate skeptic”, a broad term that collectively refers to contrarians and denialists, and also conventional scientific skeptics who are driven by more legitimate motives for dissent^28,42^. For this reason, we focus on a select set of contrarians who have publicly and repeatedly demonstrated their adamant counterposition on CC issues^12^—as extensively documented by the DeSmog project (DeSmogblog.com), a longstanding effort to document climate disinformation efforts associated with numerous contrarian institutions and individual actors.

The entry point for our large data-driven analysis is to construct a comprehensive list of adamant contrarians, which we achieved by merging multiple data sources. To be specific, we combined three overlapping sets of names obtained from publicly available sources. The first source is the list of past keynote speakers at Heartland ICCC conferences from 2008 to present; the second is the list of lead authors of the 2015 Nongovernmental International Panel on Climate Change (NIPCC) report; and the third is the list of individuals profiled by the DeSmog project. All together, we constructed a list of 386 prominent contrarians, comprised of academics, scientists, politicians, and business people who are primarily anglophone.

We then collected ∼200,000 CC research articles from the WOS database, from which we selected the 386 highest cited scientists (denoted by CCSs). These prominent scientists, many are pre-eminent CC experts with distinguished careers spanning several decades, serve as a size-balanced comparison group. Supplementary Fig. 1 lists the 100 most highly cited CCSs in our sample. We provide more detailed information on the derivation of the CCC and CCS groups in the “Methods” section and Supplementary Information Note 1.

Arriving at two lists of prominent CC actors, we then downloaded article metadata covering roughly ∼100,000 CC media articles from the MC project^37^, a public database of media article metadata (see “Methods”), which facilitates large-scale data-driven studies at the intersection of society, politics, and media^43^. For each member (hereafter distinguished by the index *i*) of the CCSs and CCCs, we counted how many media articles (given by *M*_*i*_) and in which media sources did he/she receive visibility (hereafter we distinguish media sources by the index *s*). It is important to note that MC data does not include social media posts (e.g., from Facebook and Twitter) and thus represents publicly visible hybrid content (web-only and dual print-web content) from a wide range of content producers, reflecting varying levels of production effort and quality. We also accounted for article multiplicity, i.e., articles within the MC dataset with different hyperlinks and unique article MC identifiers but with the same title and media source (see “Methods” for more details on this MC article disambiguation procedure).

**Fig. 2:**
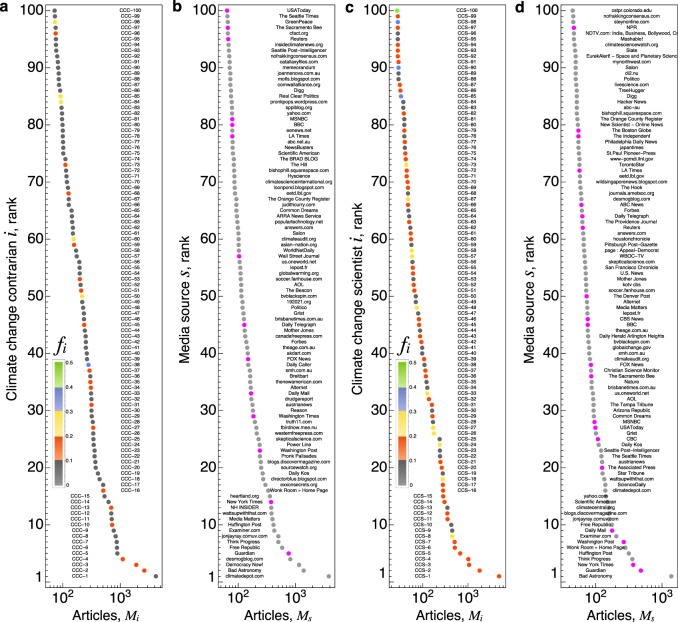
Prominent climate change contrarians (CCCs) and scientists (CCSs) in the media. **a** The 100 most-prominent CCCs in the media, ranked according to the number of Media Cloud (MC) articles; although all full names were obtained from publicly available lists, we anonymized CCC names to foster privacy. The color scale associated with each CCC indicates the fraction *f*_*i*_ of his/her articles that appear in the select-30 most prominent mainstream sources. **b** The 100 most-prolific media sources among all CCC articles. *M*_*s*_ denotes the total number of articles for a given media source, tallied across the pooled set of CCC articles. The sources colored magenta are members of the select-30 media source group. **c** The 100 most-prominent climate change scientists (CCSs) in the media, ranked according to the number of MC articles. **d** The 100 most-prolific media sources among all CCS articles

Figure 2 shows the 100 most prominent CCCs and CCSs in the media, as well as the 100 most prominent media sources. Visual comparison of the rank-ordered plots indicates significant media visibility variation both within and between the two groups. The frequency distribution *P*(*M*_*i*_) plotted in Supplementary Fig. 2a, b further illustrates the within-group variation, which is significantly right-skewed. For the CCCs, the average (median) visibility is 104 (22.5) articles; similarly for the CCSs, the average (median) visibility is 57.5 (5) articles. In contrast to these characteristic levels, the most visible individuals within each group have *M*_*i*_ in excess of 10^3^ articles. In what follows, we also leverage this within-group variation, specifically by normalizing each individual’s media visibility by their scientific achievements. This effort demonstrates the robustness of the selection criteria used to identify these two groups and the resulting compositional differences between the two groups. This broad variation points to generic success mechanisms observed in various other social systems, whereby previous achievements facilitate new opportunities, visibility, and reputation growth^44^. Yet such cumulative amplification mechanisms cannot fully explain how non-scientific experts are able to compete with scientific experts in the attention economy facilitated by the media.

### Authority in the scientific literature

We begin our comparative analysis by measuring group-level contributions to the CC literature in peer-reviewed scientific journals, namely, those that meet WOS rigorous indexing standards. In order to associate individual CCC and CCS with research articles, we match individuals’ names to the list of coauthors associated with each WOS publication, using a tested method to address the author name disambiguation problem^45^. Accordingly, we find that only 224 of the 386 CCC have a single publication in our WOS dataset. Thus, in our analysis drawing on scientific publication data, we proceed by comparing just the 224 published CCC with a size-balanced subset comprised of the 224 most-cited CCS. We denote these two subsets by 224CCC and 224CCS, respectively.

**Fig. 3:**
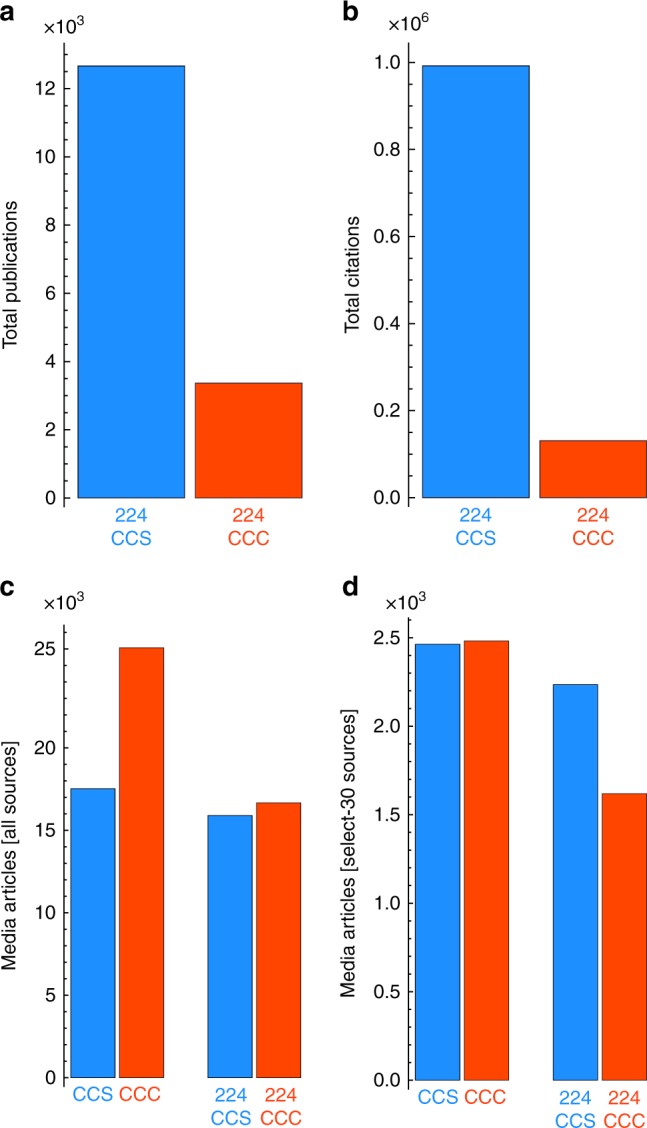
Discrepancy in scientific authority and media visibility—group level. **a** Total number of publications by the climate change contrarian (CCC; red) and climate change scientist (CCS; blue) groups. 224CCC indicates the subset of 224 CCCs comprised of just the individuals with at least one Web of Science publication; 224CCS indicates the 224 most-cited CCSs. **b** Total number of citations from the publications in **a**. Total number of unique media articles from **c** all media sources and **d** 30 select mainstream media sources

Proceeding at the group level, we tallied the total number of unique publications (i.e., counting a publication coauthored by two or more members of a given group only once). Figure 3a shows the disparity in scientific productivity, with the 224CCC subset publishing 3367 scientific articles (on average 15 articles per individual). Conversely, the 224CCS subset published 12,665 scientific articles, roughly 3.8 times more than the 224CCC.

Likewise, we tallied the total citations received by each publication set. Figure 3b shows an even larger disparity in citation impact, with 224CCS collecting roughly 7.6 times as many citations (992,206) as the 224CCC (130,833). We analyzed the degree to which this difference is larger (or possibly smaller) than what could be obtained by random chance by performing a random bootstrap sampling of the underlying productivity and citation distributions. Our simulation results show that the disparities are robust to statistical fluctuations arising from finite sample sizes and further demonstrate that the 224CCC productivity and citation impact tallies are indistinguishable from a group of CCC (see Supplementary Fig. 3).

Moreover, from a methodological perspective, the citation tally for 224CCC is likely to suffer from generous overestimation relative to the 224CCS tally (see “Methods” for further detail). To be specific, because leading researchers tend to have net citation tallies in the range of 10^3^–10^5^ WOS citations^44,46^, i.e., orders of magnitude greater than the citations accrued by the average papers in their field, the misattribution error associated with name ambiguity only marginally increases the citation tally *C*_*i*_ for elite scientists belonging to the 224CCS group; contrariwise, misattribution error could significantly increase *C*_*i*_ for the majority of the 224CCC group members. Thus the 660% difference in group-wise citations is a lower-limit estimation of the disparity in scientific authority between these CCS and CCC.

### Visibility in the media

We continue by comparing group-level media visibility across a large collection of digital and print media CC articles collected from the MC project^37^. Much like the WOS publication data, which is derived from various journals, our media article data is derived from a wide range of media sources—including print newspapers and magazines, as well as online media (e.g., online news sites, personal and society blogs). Figure 2 shows the most prominent individuals and media sources associated with the CCC and CCS groups, respectively.

We performed two group-level comparisons, one using all media articles and one using a subset of media articles coming from 30 select media sources (see Supplementary Fig. 4 and Supplementary Table 1). These select-30 media sources account for 11% (11,233 articles) of the total media articles analyzed. Because the select-30 media source set was chosen manually, we also compared article counts per group using media source groups identified by an automatic clustering algorithm, which yields consistent results (see Supplementary Fig. 5).

Tallying across all media sources, we count 26,072 articles for CCCs, roughly 49% more than the 17,530 articles associated with CCSs (see Fig. 3c). Tallying across just the select-30 sources, we obtain nearly equal counts: 2482 articles for CCCs and 2463 articles for CCSs, corresponding to just a 0.77% excess for CCCs (see Fig. 3d). Upon further inspection, we found that these count differences also strongly depend on the underlying group composition—in addition to the composition of the underlying media sources.

Repeating the comparison using just the subsets of 224 academically oriented individuals, we obtain a negligible difference of just 1% (16,670 articles for 224CCC and 15,896 for 224CCS). Thus conditioning on either visibility in the mainstream media or on visibility by academically oriented individuals yields parity. However, proceeding with the comparison conditioned simultaneously on select-30 sources and academically oriented individuals reveals a 38% media visibility advantage in favor of the elite scientists (1619 articles for 224CCC and 2235 for 224CCS). These results highlight the nuances associated with comparing groups comprised of individuals with fundamentally different professional orientations. Yet even in this latter and most relevant case, where we compare 224CCC and 224CCS in the mainstream media, there still remains a remarkable discrepancy in the scientific authority and media visibility between these more academically oriented scientists and contrarians.

To further distinguish visibility in the mainstream media, as opposed to blogs and other new media sources, we calculated the fraction *f*_*i*_ of articles associated with each individual published by each media source belonging to the select-30 media source group. The color gradient in Fig. 2a, c indicates the value of *f*_*i*_ for the most prominent individuals, revealing how contrarian visibility from mainstream media sources is more concentrated on a relatively small CCC subset. To facilitate group-level comparison, we also calculated the distribution *P*(*f*_*i*_) for individuals with *M*_*i*_ ≥ 10 articles (in order to eliminate individuals with large *f*_*i*_ due to small sample size fluctuations). Comparison indicates that, among these more prominent individuals, the average CCS has roughly twice the mainstream prominence as the average CCC; the distribution *P*(*f*_*i*_) for the CCSs is also more right-skewed than for the CCCs, see Supplementary Fig. 2c, d. While these results may appear to be inconsistent with the group-wise totals shown in Fig. 3c, d, this apparent discrepancy arises from the fact that multiple CCSs and CCCs can be associated with the same MC article.

Together, these results show that CCCs derive a comparative visibility advantage from non-scientists gaining attention in peripheral non-mainstream media sources. Conversely, the observed parity between CCCs and CCSs in mainstream media sources may reflect media writers seeking journalistic balance when reporting on CC. Indeed, we find that every select-30 media source has provided CCC significant visibility, thereby increasing CCC authority and credibility (see Supplementary Fig. 4). The disproportionate visibility of CCCs, even in mainstream media sources, is reminiscent of early contrarian efforts that leveraged the U.S. Federal Communications Commission Fairness Doctrine to obtain equal press time^6^. While this policy was officially discontinued in 1987, journalists may still be using it to justify mentioning and interviewing counterpositions when writing on contentious issues such as CC. Indeed, communication scholars have noted that, in the case of CC, such disproportionate visibility—or false balancing—is likely to mislead public perception, suggesting falsely that within the scientific community there is parity in the number of scientists who do and do not agree on the fundamental issues of anthropogenic CC^6,8–12^.

### Juxtaposing authority and visibility at the individual level

**Fig. 4:**
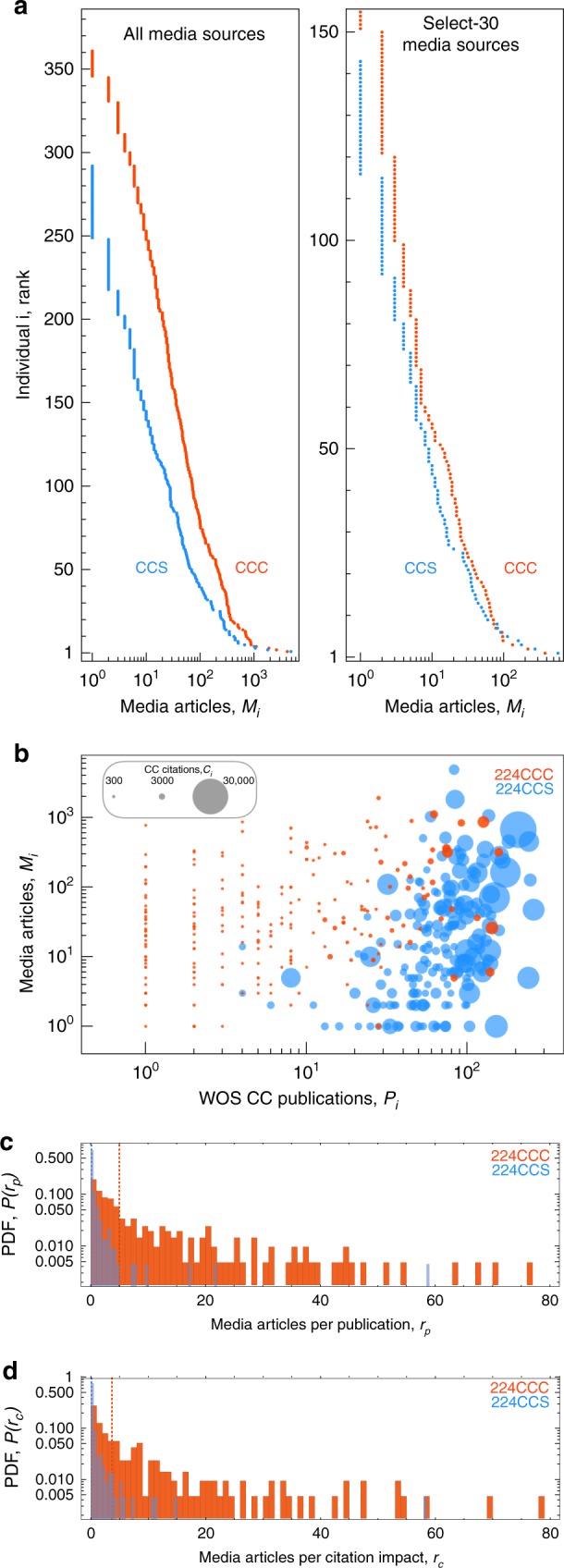
Discrepancy in scientific authority and media visibility—individual level. **a** Individuals ranked by their number of media articles, *M*_*i*_. (right) *M*_*i*_ calculated using select-30 mainstream media sources only. **b** Scatter plot of individuals comparing Web of Science publications *P*_*i*_ versus media visibility *M*_*i*_; point size is proportional to the log of total citations, ln* C*_*i*_. **c** Probability distribution *P*(*r*_*p*_) of media visibility per publication, *r*_*p*_ ≡ *M*_*i*_/*P*_*i*_; vertical dashed line indicates the distribution median. **d** Probability distribution *P*(*r*_*c*_) of visibility per citation impact, *r*_*c*_

To test whether the discrepancy in scientific authority and media visibility is also present at the individual level, and not the result of just a few outliers driving group totals, we disaggregated the WOS and MC data into individual profiles. Figure 4a compares the article count *M*_*i*_ between individuals of the same rank within their respective groups. CCCs are consistently more visible in the media relative to their CCS counterparts; this disparity persists even when comparing visibility within the prominent select-30 media sources.

Figure 4b shows the total number of media articles (*M*_*i*_), the total number of WOS publications (*P*_*i*_), and the total number of citations (*C*_*i*_). Shown together, this representation highlights the relatively small intersection between the two groups. Despite CCCs holding advantage in gross media visibility, just a few CCCs are on par with the scientific achievement of career experts. Moreover, the scatter plot indicates that CCCs are more likely to have larger *M*_*i*_ values than their CCS counterparts within the same *P* range. Thus, despite the selection criteria that explicitly gives CCSs the advantage in the scientific domain, the discrepancy between the two groups is manifestly prominent.

To further emphasize this point, we also calculated the visibility per unit of scientific achievement, thereby accounting for compositional differences between the two groups at the individual level. The ratio *r*_*p*,*i*_ ≡ *M*_*i*_/*P*_*i*_ measures the number of media articles per publication for each individual. Similarly, $r_{c,i} \equiv M_i/\sqrt {1 + C_i}$ measures the media articles per citation impact, where the square root is used to adjust for the skew in *C*_*i*_, while *C*_*i*_+1 avoids the singularity for individuals with *C*_*i*_ = 0. Figure 4c, d show the probability distributions, *P*(*r*_*p*_) and *P*(*r*_*c*_), which convey the markedly different ranges and concentrations of *r* between the 224CCC and 224CCS. For both measures, the mean (respectively median) *r* value calculated for 224CCC is ∼15 (∼40) times larger than the mean (median) value for 224CCS. However, distributions calculated using ratio values scaled by the group average (e.g., $\tilde{r}_{p,i} \equiv r_{p,i}/\langle {r_p} \rangle$) indicate a common distribution shown in Supplementary Fig. 3d, e.

This distribution scaling result indicates that the mean media visibility per scientific authority is an appropriate group-level indicator. As such, if we use the 162 CCSs not included in the 224CCS group as an alternative comparison group, one that is comprised of scientists who are not nearly as elite as the 224CCS, we still find that the 〈*r*〉 values for this alternative group are on par with the average values for 224CCS: The group mean values are 〈*r*_*p*_〉 = 15.4 (224CCC), 1.04 (224CCS), 1.66 (162CCS); 〈*r*_*c*_〉 = 18.8 (224CCC), 0.94 (224CCS), 0.47 (162CCS). Thus, by controlling for individual-level variation in scientific authority, we show that, even compared to a less prominent scientific comparison group, the 224CCC still have remarkably high media visibility per scientific authority.

### Classification of how individuals are sourced in the media

In order to identify how CCCs and CCSs obtain media visibility, we analyzed the full-text content of 2256 media articles produced by 6 mainstream media sources: the Guardian, New York Times (NYT), Washington Post (WP), FOX News (FOX), LA Times (LAT), and the Wall Street Journal (WSJ). For each article, we located individuals’ names and inferred the context associated with their sourcing, which we annotated according to five types (see “Methods” for further details). In order to facilitate comparison, we further grouped these five types into two broad categories: mentioned, representing a passive sourcing; and contributed, representing a more active sourcing.

**Fig. 5:**
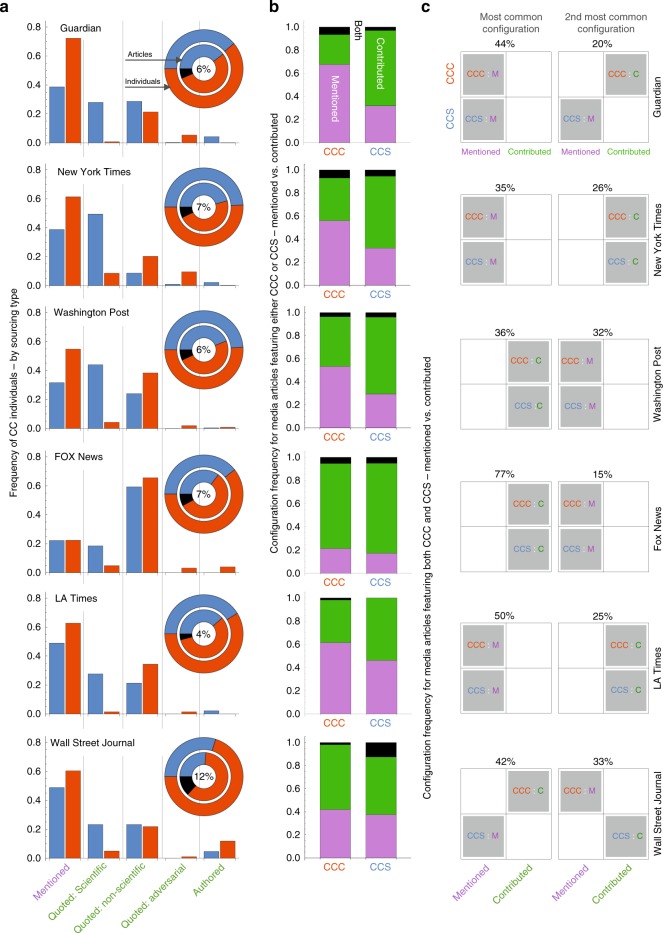
How climate change contrarians (CCCs) and climate change scientists (CCSs) are sourced in CC articles—by media source. **a** Frequency distribution showing how individuals are sourced in media articles according to five types separated into two categories: mentioned (purple) and contributed (green). Pie-chart insets: The outer ring indicates the fraction of individuals analyzed by group; the inner ring indicates the fraction of articles analyzed featuring individuals from just CCCs (red arc), just CCSs (blue arc), or both groups (black arc, with this percent value indicated at the center). **b** The frequency of three sourcing configurations for unbalanced articles featuring either CCCs or CCSs but not both. The black segment indicates the frequency of articles featuring mentioned and contributing individuals. **c** The two most common sourcing configurations for balanced articles featuring both CCCs and CCSs. For example, articles with CCCs and CCSs mentioned is the most frequent configuration for The Guardian, which occurred in 44% of the articles featuring both CCCs and CCSs; the second most frequent configuration featured CCCs contributing and CCSs being mentioned (20%)

Figure 5a shows the frequency of each sourcing type by media source, revealing mentioned as the most common sourcing type. The main exception is FOX, which tends to quote individuals on non-scientific grounds, which is also fairly common in the WP. Another common sourcing are quotes containing scientific content, which are more common for CCSs, as CCCs are rarely associated with this sourcing type. The least common sourcing types observed are non-scientific quotes and adversarial quotes, with most instances of these types associated with CCCs. Notably, the Guardian, NYT, and WSJ featured the most number of articles authored by CCSs.

By focusing our content analysis on individuals, we are able to estimate the frequency of cross-group articles—those articles that source both CCCs and CCSs. Our analysis indicates cross-group articles to be around ∼7% for each source, with the exception of the WSJ, which featured both groups in 12% of its articles. These percentages are likely to be a lower-bound estimate to the frequency of balanced sourcing of individuals from each group within the same article, since it is also possible that individuals not included in our select sets of 386 individuals were also mentioned or contributed to these articles. As a result, the most common configuration we observed features just CCCs or just CCSs but not members of both groups. Figure 5b indicates the relative frequency of the three possible sourcing configurations, showing that articles featuring just CCCs (respectively just CCSs) are those with individuals classified as mentioned (respectively contributed); the exceptions are FOX and WSJ, which instead most commonly feature CCCs as contributing sources. This is consistent with previous analysis of FOX, which observed a greater ratio of CC doubters to believers among those interviewed, as compared to CNN and MSNBC^47^.

A common theme in the CC communication literature is false balance, representing how the journalistic tradition of balancing sources across opposing views gives rise in the case of CC to an inaccurate representation, one that falsely suggests that there is a balanced debate between equally sized groups^8,9,11,12^. Figure 5c provides insight into this phenomena by showing the two most common configurations for the subset of articles featuring both CCCs and CCSs. Our results show that the most common motif among articles sourcing CCCs and CCSs are those that are also balanced by sourcing type, with the exception of the WSJ, which instead tends to include CCC contributions juxtaposed by CCS mentions. Among the sources that balance according to sourcing type, the Guardian, NYT, and LAT most commonly mention individuals from each group, whereas WP and FOX tend to incorporate individual contributions from each group.

### Co-visibility in the media

It was unfeasible to apply the content analysis to the entire dataset, and so we turn to network analysis to identify additional relational patterns of co-visibility within groups and across their media interface. To proceed, we first merged the sets of CCS and CCC media articles. Whereas *M*_*i*_ counts the total number of media articles for individual *i*, the co-visibility *M*_*ij*_ ≤ *M*_*i*_ counts the number of articles that feature both individuals *i* and *j*. Combining the matrix elements *M*_*ij*_ calculated for all pairs of individuals, we construct the symmetric co-visibility matrix **M**. Supplementary Fig. 6 shows **M** calculated in two ways: using all media sources and using just the select-30 media sources. Note that individuals with *M*_*i*_ = 0 are not included in the matrix **M**; and for an individual with *M*_*i*_ > 0, if they do not appear in any media articles with any other individuals, then they are also not included in **M**.

Visual inspection of the co-visibility matrix reveals two fundamental features. First, CCCs are more prominent than CCSs when two or more individuals are featured in the same media article: 58% of individuals who have appeared in a media article with another individual are CCCs; considering co-visibility within select-30 media sources, this visibility advantage grows to 62%. Second, the strongest co-visibility (largest *M*_*ij*_) are within group rather than between group (see Supplementary Note 2 and Supplementary Fig. 6), reflecting the results of our content analysis.

**Fig. 6:**
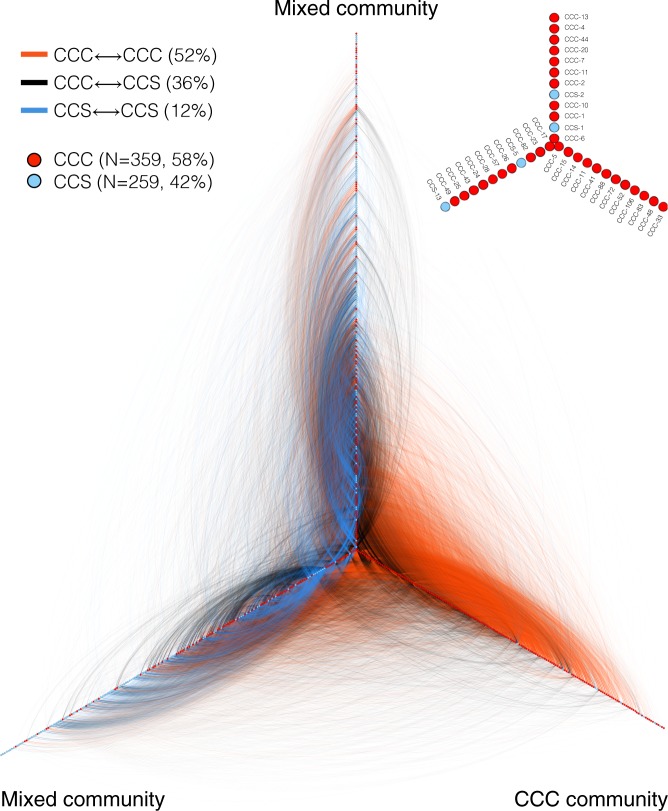
Media article co-visibility network—individual level. Clustered representation of the co-visibility network: nodes are climate change contrarians (CCCs) and climate change scientists (CCSs) who have at least one media article associated with at least one other individual. Links are colored according to three types: links between members of the CCC (CCS) group are red (blue) and links between groups are black; the percentages of links by type are reported in parentheses (e.g., 52% of links are within the CCC group). We used a modularity-maximizing clustering algorithm^48^ to identify three communities, with nodes ordered along each spine according to its network centrality—as such, the most prominent individuals are located at the apex. Two communities are well mixed, whereas the third represents an extremely polarized echo chamber comprised primarily of CCC. (inset) Magnification of the apex showing the most prominent individuals

To illustrate this latter point, we applied the Louvain modularity maximizing algorithm^48^ to cluster the co-visibility matrix into communities of individuals. To be specific, we applied this unsupervised algorithm to identify groups of individuals who are more connected to other individuals within their community than without. Figure 6 uses a network visualization layout in which communities are indicated by each network spine, revealing a three-community structure. Moreover, we ordered the individuals (nodes) along each spine according to their network centrality (using the PageRank metric), such that the most prominent individuals within each community are located toward the apex. Inspection of the composition of each community reveals two types: two are mixed and the third is primarily composed of CCCs—a clear example of an archetypal echo chamber.

### Asymmetric flow of citations within the CC citation network

We also analyzed the organizational patterns recorded in the WOS citation network. Citation networks reconstructed from the reference lists of publications provide insight into the evolution of the scientific endeavor—a complex system emerging from the interactions between researchers, scholarly outputs, collective knowledge, and emergent culture^49^. Scientific authority, which emerges from the repeated interactions of individuals within the community of active scientists, can thus be inferred from citation totals at varying levels of aggregation^44^. In the present context, distinct citations represent quantifiable interactions between individuals, likely ranging from attribution, to critique, to outright dismissal. This latter type of negative citation occurs relatively frequently^50^, reflecting the oppositional nature of debate around contentious scientific issues^51^.

**Fig. 7:**
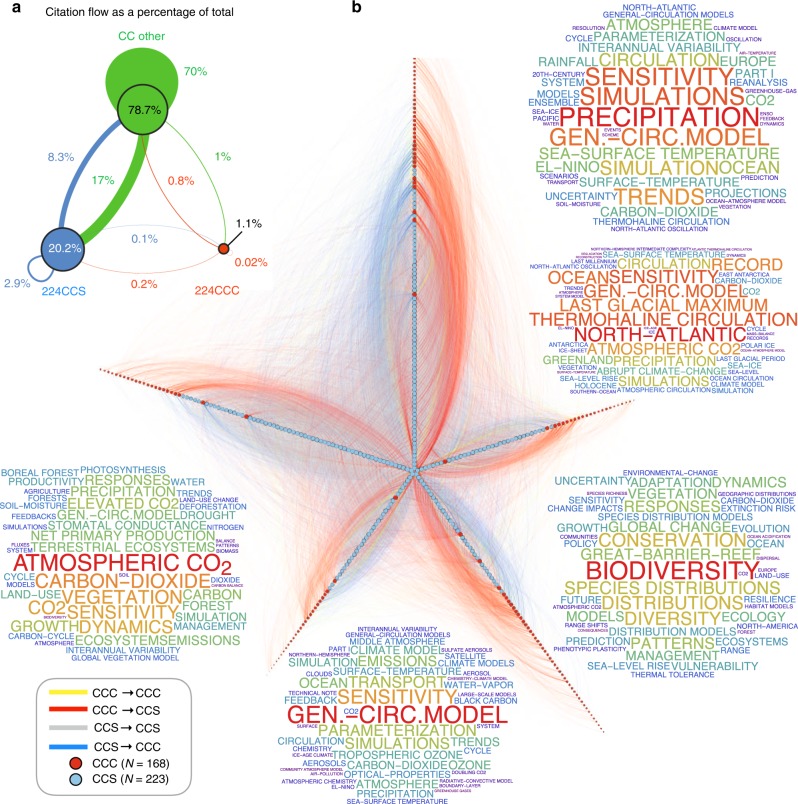
Research article citation network—group and individual level. **a** Within-group and between-group citation flow as a percentage of the total number of citations produced across three researcher groups. Node size captures the net citation flow into a given group; link width is proportional to the fraction of the total citation flow, with link color indicating the source group. For example, 20.2% of the total citations are directed toward 224 climate change scientists (CCS) (corresponding to 0.44% of the total 50,442 researchers analyzed in the group-level citation analysis), whereas only 1.1% are directed toward the 224 published climate change contrarians (CCC); roughly 17 times as many citations flow from the CC Other to CCSs as from the CC Other to CCCs. **b** Nodes in the network are CCS and CCC researchers with at least one publication receiving at least one citation from another node (i.e., connected within the citation network); roughly 90% of the nodes are CCSs because 218 CCCs have no publications citing or cited by other publications within the set of publications by CCCs and CCSs. The links capture the total number of citations flowing from publications authored by scientist *i* to the publications authored by scientist *j* and are colored according to the source node; gray links are de-emphasized using low opacity level. Node size is proportional to the log of the total edge weight (citations) entering a given node. We used the Louvain modularity-maximization method^48^ to identify groups of nodes belonging to a particular community—i.e., groups of node that are more connected to other nodes within the cluster than without. These communities are plotted along each of the spines, with nodes ordered according their size, so that the most prominent individuals are located at the center. Each community contains several CCCs, located mostly at the peripheral (low-prominence) tips, with just a few exceptions. Word clouds show the 50 most frequent Web of Science publication keywords associated with each community; keyword size is proportional to the log frequency

In this way, we used the CC citation network to assess the flow of authority between the two research-oriented subsets, 224CCC and 224CCS, at both the group and individual level. We start at the group level, using the ∼50,000 other CC scientists who were not members of either the 224CCC or 224CCS groups as an external self-consistent comparison group. Figure 7a shows the proportion of CC scientific article citations flowing between the 224CCC, 224CCS, and CC Other groups, with 224CCS having 17 times the citation authority as 224CCC (20.2% of the total citations from CC Others are directed toward 224CCS, whereas 1.1% are directed toward 224CCC). In direct comparison, the 224CCC cited 224CCS twice as often as in reverse. Even after normalizing citation rates by group productivity, we find that 224CCC cite 224CCS 20 times more frequently than in reverse and that 224CCS receive 79% more citations than they produce (see Supplementary Fig. 3a).

We also analyzed the directed citation flow between any given pair of individuals that occurs when a publication *p*_*a*_ authored by individual *a* cites a publication *p*_*b*_ authored by individual *b*. To be specific, we counted the citation linkages *a* ∼ *p*_*a*_ → *p*_*b*_ ∼ *b* that connect any pair of authors, where *a* ∼ *p*_*a*_ indicates that individual *a* is an author of publication *p*_*a*_, and *p*_*a*_ → *p*_*b*_ indicates that publication *p*_*a*_ cited *p*_*b*_.

Figure 7b shows the resulting interpersonal CC citation network, comprised only of scientists who gave or received at least one citation within our WOS dataset. As such, there are only 168 CCCs connected within this citation network. We again used the Louvain algorithm^48^ to identify groups of nodes that are densely connected together, representative of coarse research communities. Our results show five communities, each represented as a spine with individuals ranked according to centrality, with the most authoritative individuals located at the apex. Represented as such, the network of scientific authority shows that the majority of CCC are located toward the periphery. Interestingly, the peripheral CCC within each community appear to direct most of their citations toward the most prominent CCS, possibly representing adversarial interaction in the form of negative citations aimed at discrediting their research findings^50^.

## Discussion

CC is a wicked multidimensional problem, whereby individual dimensions—i.e., environmental, socio-economic, technological, science communication—while separately challenging, together pose the 21st century’s pre-eminent grand challenge. In this regard, a public that is unaware of the realities and risks associated with CC poses a threat to society and planet by undercutting strenuous global efforts to rapidly mitigate threats to the planet’s biosphere.

Understanding Earth’s coupled human–environmental systems requires broad and deep knowledge of processes occurring across a range of scales—from microscopic chemical processes to macroscopic thermodynamic flows and human consumption and land-use trends that span the entire global system^52^. The monumental task of drawing together and integrating expertise across numerous research domains will require intense trust-based collaboration across disciplinary, organizational, and political boundaries^35^. To this end, the consortium science framework^53^—whereby teams of teams organize around a common goal, with a mission to share returns both within and beyond organizational boundaries—is an appropriate model for facilitating cross-disciplinary knowledge exchange and achieving the transformative breakthroughs needed to address this grand challenge. We see this collaborative pattern in the structure of citations within the broader CCS community documented here (Fig. 7), but not within the CCC community, which is too small to encompass the complexity required to grapple with the fundamental issues of CC science. At the same time, this complexity poses a significant challenge to communicating climate science to the broader public, which makes the public discourse on climate science more vulnerable to the opinions of contrarians whose prominence in the media is disproportionate to their representation in the scientific community.

Indeed, communicating authoritative information about the risks of inaction is crucial for achieving global action. Yet, sending uniform and authoritative messages is challenging for various reasons. One reason is that CC communication often requires strategically paring down this wicked problem for non-expert audiences^54^. A related problem is the diminishing demand for expertise in scientific discourse aimed at the public^40^. These problems are further exacerbated by the proliferation of new media, which democratize the production and consumption of information, making it increasingly challenging to identify trustworthy information^55^. Unless countered by improvements to quality control management that can match the production scale, this information deluge is likely to overwhelm the traditional safeguards of professional editorial oversight.

Against this background, we contribute to the CC communication literature on authoritative messengers and new media by analyzing two carefully selected groups of prominent individuals who are frequently sourced in CC media articles. In order to operationalize this individual-centric approach, we located the digital footprints of 772 individuals in ∼200,000 research publications indexed by the WOS and ∼100,000 media articles produced by both traditional and new media sources based primarily in North America and Europe. Thus, by juxtaposing media visibility and scientific authority for two counter-positional groups, we are able to objectively measure the discrepancy in CC authority between consensus scientists (CCS) and contrarians (CCC). In particular, by contrarians we refer to individuals frequently sourced by institutions denying the documented realities of CC and its consequences and/or individuals who have personally expressed inaccurate statements. As such, we selected CCC using open registries that clearly document their contrarian positions.

There are several limitations to our data-driven analysis worth first discussing. First, we do not account for the range of professional backgrounds, nor do we account for the different types of skepticism promoted by different CCC^28^. By way of example, recent work comparing fundamental skepticism (relating to sources and existence of CC) to impact skepticism (relating to potential impacts of CC) reveals that the frequency of the fundamental skepticism has decreased over time, whereas the frequency of impact skepticism has increased over time, possibly signaling a strategic shift within the contrarian movement^33^. While distinguishing visibility according to these two skeptic types could explain some variation in media visibility observed across individuals (Fig. 2), in-depth content analysis to assess individuals’ relative positions on CC was not feasible nor the aim of our study but rather serves as an avenue for future research.

A second limitation relates to the sampling of fixed group sizes of 386 individuals from global populations of contrarians and scientists who differ greatly in their size. As such, the disparity in visibility may be affected in part by there being relatively fewer (greater) number of contrarians (scientists) combined with journalists seeking balance^8,9,11,12^. Another source of variation that limits the interpretation of our comparison is the composition of the CCC group, which includes business people and politicians in addition to skeptic scientists, thereby reflecting the same drivers of variation underlying the primary frames (e.g., science, culture and society, political economics, and environment) identified within a corpus of newspaper articles on CC^36^. We addressed this compositional difference in several ways. First, we restricted our analysis to the time period before the 2016 US presidential election so that media visibility is more reflective of the scientific rather than the political arena; second, we focused our analysis of scientific authority drawn from peer-reviewed research on the subset of 224 CCCs who did appear in the publication data, and compared them with a size-balanced set of 224 CCSs; and third, we compared the two groups using normalized media visibility measures in order to account for variation in scientific authority between the 224CCC and 224CCS (Fig. 4). In this way, we explored alternative explanations for observed discrepancies in visibility and authority. One final limitation relates to how individuals appear in media articles, as we do not distinguish whether individuals are sourced as experts or dismissed as illegitimate authorities^12^. Thus, as in the case of positive and negative citations, our measures of media visibility are partially conflated by dismissive mentions.

While the size of the contrarian movement may be relatively small, our study reveals the degree to which new media facilitates the production and mass distribution of assertive content by CCC—which intentionally or not, crowds out the authoritative message of real CCS. To be specific, tallying across all media sources we find CCC media visibility to be 49% greater than CCS visibility. However, if we condition the article count tallies using select mainstream media sources, i.e., sources that implement quality control through more traditional editorial standards [Supplementary Table 1], the media visibility of the two groups is remarkably on par. Only when comparing the visibility of the academically oriented subsets of 224CCC and 224CCS in mainstream media sources do the elite scientists in our study establish a marked visibility advantage (38%)—despite the remaining 280% (publications) and 660% (citations) disparity in scientific authority (Fig. 3). As such, we objectively demonstrate the discrepancy in the scientific authority and media visibility between these two sets of prominent CCS and CCC.

In order to provide contextual depth, we also analyzed how these prominent individuals are sourced within media articles—are they just mentioned or do they contribute content via quotes or authorship? This assessment involved full-text analysis of ∼2,000 media articles from 6 prominent media sources: Guardian, NYT, WP, FOX, LAT, and the WSJ. Our results point to an additional level of discrepancy—CCCs are more likely than their counterparts to be mentioned or contribute via non-scientific quotes, whereas CCSs are more likely to contribute via scientific-oriented quotes or authorship. FOX News and the WSJ are two exceptions to these patterns, as they tend to source quotes and authorship contributions from CCCs and CCSs more equally. Research shows that journalists often quote contrarians either to infuse objectivity or to dismiss their position outright^9,12^. Yet, these approaches also detract attention from the relevant CC narrative and provide the counterproductive impression that there is something substantial in contrarian arguments to be debated. Thus the time has arrived for professional journalists and editors to ameliorate the disproportionate attention given to CCCs by focusing instead on career experts and relevant calls to action.

Our individual-centric approach also provides insight into the structural properties of the consensus–contrarian interface. Analysis of the interpersonal citation network reveals that 224CCS are cited by the remaining CC community 17 times more frequently than 224CCS. Even accounting for productivity differences, we find that 224CCC cite 224CCS 20 times more frequently than in reverse. Similar analysis of the media co-visibility network shows that 52% of the associations are between CCCs, whereas only 12% are between CCSs, an additional disparity pointing to manifest organizational differences, such as the contrarian echo chamber illustrated in Fig. 6.

In summary, our work contributes to recent computational social science efforts^7,34,38,49,56^ by leveraging new opportunities in large-scale data collection and analysis^37^ to clarify both the individual and collective properties of complex socio-technical systems^57^. Related research on the emergence of polarization around critical yet controversial socio-political issues^51,58^, the impact of new media on the public^59^, and the spread of inaccurate information^16,17,43^ will together provide important guidance on how to improve the effectiveness of CC communication^14,15,21,32,60,61^. Indeed, despite the challenges posed by new media, there are also new opportunities. One relevant example that borrows from the post-publication peer-review system in science is the new media tool climatefeedback.org^62^, which allows expert scientists the opportunity to annotate, grade, and correct inaccurate CC information published in the media^63^.

## Methods

### MC data

We collected a dataset of 121,729 unique print articles, online articles, and blog posts on CC from 01 January 2000 to 01 October 2016 from 7126 unique media sources from the MC project, an open data project hosted by the MIT Center for Civic Media and the Berkman Klein Center for Internet & Society at Harvard University (https://cyber.harvard.edu/research/mediacloud). See Supplementary Note 2 for additional details on the data collection.

We chose to set the upper bound for the MC data collection as October 2016 so to avoid confounding the deluge of CC articles related to US elections and subsequent cabinet and other government administration appointments. One clear limitation of our MC dataset is that it is biased toward English-language content (98.6% of articles analyzed are classified as English language by MC); however, since the discourse on CC began in the scientific domain, where English is also the de facto communication language, we do not believe this regional bias significantly affects our comparative results within the domain of English-based science and media content.

We then refined the dataset by applying the following media article disambiguation method. Upon close inspection of individual MC article metadata, we found that a significant number of articles indexed by MC refer to the same media article. We found this to be particularly common among the select-30 media sources included in our content analysis, in which the same article may have multiple different URLs, representing an array of hyperlinks from different facets of their website—e.g., blog section, RSS feed section, and front page—to a common media article. As such, the initial set of 121,729 MC articles requires an additional merging procedure in order to avoid overcounting, resulting in a final dataset of 102,250 unique MC articles.

While two or more MC articles may have unique MC article identifiers, inspection of the URL and article title indicate that they are indeed the same. By way of example, consider the following MC articles: the first is http://www.nytimes.com/2016/05/20/science/exxon-mobil-climate-change-global-warming.html?partner=rss&emc=rss, with MC title *State Officials Investigated Over Their Inquiry Into Exxon Mobil’s Climate Change Research* and MC unique identifier 468593919 (the unicode HTML encoding for the apostrophe (') symbol is intentionally left uncleaned in order to illustrate challenges in excess of simple string matching); and the second is http://www.nytimes.com/2016/05/20/science/exxon-mobil-climate-change-global-warming.html, with MC title *State Officials Investigated Over Their Inquiry Into Exxon Mobil’s Climate Change Research* and MC unique identifier 468588600.

We addressed this redundancy by merging articles with the same MC media source and similar title into a single article instance so that article counts for sources and individuals are not systematically inflated. We determine whether two titles are similar by calculating the Damerau–Levenshtein edit distance *D*_*jk*_ between the title *T*_*j*_ and *T*_*k*_. Titles that are similar have small *D*_*jk*_, meaning that a small number of character insertions, deletions, and swaps can convert *T*_*j*_ and *T*_*k*_, or vice versa since it is a symmetric measure. Thus we merged two articles if *D*_*jk*_ ≤ 0.2 × Min.[|*T*_*j*_|,|*T*_*k*_|], where |*T*_*j*_| is the string length of title *j* after removing all HTML encodings and ASCII control codes and extended characters that are present in the title—i.e., first refining the title *State Officials Investigated Over Their Inquiry Into Exxon Mobil’s Climate Change Research* into *State Officials Investigated Over Their Inquiry Into Exxon Mobils Climate Change Research*. We calculated *D*_*jk*_ between all pairs of MC articles from the same media source, which refined the total dataset size from 121,729 to 102,250 MC articles, a 16% reduction. We observed similar reduction levels for the set of articles belonging to each CCC and CCS group.

### CC publication data

We manually collected a dataset of 198,789 articles from 1900 to 2016 derived from the WOS Core Collection database (https://apps.webofknowledge.com/) using the search query (Climate NEAR/5 Change OR Global NEAR/5 Warming OR Climatology OR Climate NEAR/5 Model OR Climate NEAR/5 Extreme) AND (Climate); this query identifies a broad set of research streams relating to CC, while reducing the inclusion of more general meteorology and environmental research that refers to climate but not in the particular context of CC. Supplementary Fig. 1a shows the topical space of this WOS dataset on CC, confirming that our query captured the broad range of publications associated with the highly multi-disciplinary domain of CC research.

### Selection of contrarians (CCC)

We compiled a list of 386 contrarians by merging three overlapping name lists obtained from three public sources. The first source is the list of former speakers at The Heartland Institute ICCC conference (http://climateconferences.heartland.org/speakers/) over the period 2008–present, providing a representative sample across time; the second source is the list of individuals profiled by the DeSmogblog project; and the third source is drawn from the list of lead authors of the most recent 2015 NIPCC report (the principal summary of CC denial argumentation produced in conjunction with The Heartland Institute, http://climatechangereconsidered.org/).

### Selection of scientists (CCS)

We ranked individuals’ publication profiles according to the net citations $C_{i} = \sum_{i \in p} c_{p}$ calculated by summing individual article citation totals (*c*_*p*_) for only the individual articles (indexed by *p*) included within our WOS CC dataset. In this way, the CCS group is comprised of the 386 most-cited CC scientists, based solely on their CC research.

### Author name ambiguity measurement error

Because we are comparing the most prominent members from each group, we assume that each individual has already made his/her dominant mark in CC research, and so the measurement bias arising from comparing researchers of different ages and citations from different time periods should not significantly affect the group-wise comparison. Another source of error occurs in the attribution of publications to authors commonly known as the author name disambiguation problem. We used a tested initial-based author name disambiguation approach^45^, which nevertheless tends to overestimate the number of publications for a given author, thereby corresponding to a positive misattribution or clumping error. Since not all CCC are career academics or research scientists, this misattribution error tends to artificially boost an unpublished individual’s total number of publications *P*_*i*_ from 0 to *P*_*i*_ ≡ *η* ≥ 0, where *η* is a random variable representing misattribution noise. Since CCSs are by construction experts in the scientific community with *P*_*i*_ ranging from 10^2^ to 10^3^, the relative error *η*_*i*_/*P*_*i*_ is significantly smaller for CCSs than for CCCs. It follows that misattribution noise (*η*) should affect both groups the same in absolute magnitude, since the group sizes are balanced; however, as a relative percentage, this error inflates the CCC tallies more than the CCS tallies. See Supplementary Note 1 for additional discussion of our author name disambiguation strategy.

### Select mainstream media sources

Analysis of the individual media sources producing CC content also revealed a wide range of production volume. To account for this variation, in what follows we distinguish media visibility within 30 prominent mainstream sources. The members of the select-30 group, ordered according to the total number of CC articles analyzed (in parenthesis), are: the Guardian (1949), New York Times (1188), Washington Post (854), Daily Mail (806), Reuters (473), FOX News (431), Daily Telegraph (406), Washington Times (387), The Sacramento Bee (380), MSNBC (354), Associated Press (298), LA Times (287), Time (287), USA Today (285), Independent (260), The Denver Post (253), Wall Street Journal (244), BBC (243), Miami Herald (243), ABC News (235), CBC (212), Boston Globe (209), CNN (202), CBS News (193), NPR (146), Chicago Tribune (112), Globe and Mail (108), Deutsche Welle (92), NBC News (51), and Seattle Times (45). Importantly, the members of the select-30 were chosen independent of CCCs and CCSs. Supplementary Fig. 4 shows the fraction of each media source’s articles featuring just CCCs, just CCSs, and both groups simultaneously. Supplementary Table 1 lists additional information about the type, age, MC unique identifier, and number of unique MC articles collected from each media source.

### Content analysis of full-text articles

We manually located the full-text of each article using the hyperlink provided by MC and then tallied how individuals were sourced according to a typology comprised of five categories. The first class corresponds to individuals who are mentioned—but not actively involved in the article content. The second corresponds to individuals quoted—and including scientific content. The third corresponds to individuals quoted—but not including scientific content. The fourth corresponds to a scenario similar to the previous category, in which individuals are quoted—including adversarial content aimed at the counterposition. And the fifth corresponds to when the individual authored the media article.

The first category represents a reference to an individual, often related to a documented event such as a report or conference, that does not include any attributable content. As such, it is likely that individuals sourced in this way did not actively contribute to the production of the media article, and so these mentions do not necessarily confer any privileged expert status upon the individual. However, the remaining four categories reflect some level of contribution by the individual—either active in the case that they were interviewed or passive in the case that statements were quoted from external text, e.g., from a research publication. In the case of quoted categories (2–4), we do not distinguish between quotes derived from external sources (e.g., media articles, reports, or scientific publications) or quotes derived from personal interview. We then group sourcing categories (2–5) together into a combined contributed category to facilitate direct comparison with the mentioned category. This approach follows a framework for evaluating references to the IPCC and non-select contrarians in the media^12^. We also chose this schema because it is readily identifiable, generalizable to different media source types, e.g., daily newspapers (Guardian, NYT, WP, LAT, WSJ) and news cable (FOX).

## Supplementary information

Supplementary Information

## Data Availability

All data analyzed here are openly available from Web of Science and the Media Cloud project. Supporting article- and individual-level data are available at the UC DASH data repository^64^.

## Code availability

Code used to carry out media source analysis in this manuscript is available along with the data in ref. ^64^. Reasonable additional requests and questions about code can be directed to A.M.P.
