# Author Correction: Discrepancy in scientific authority and media visibility of climate change scientists and contrarians

**DOI:** 10.1038/s41467-019-12061-4

**Published:** 2019-08-29

**Authors:** Alexander Michael Petersen, Emmanuel M. Vincent, Anthony LeRoy Westerling

**Affiliations:** 1 Management of Complex Systems Department, Ernest and Julio Gallo Management Program, School of Engineering, University of California, Merced, CA 95343 USA; 2 Medialab, Sciences Po, Paris, 75007 France; 3 Center for Climate Communication, University of California, Merced, CA 95343 USA; 4 Sierra Nevada Research Institute, University of California, Merced, CA 95343 USA

**Keywords:** Computational science, History, Climate change

Correction to: *Nature Communications* 10.1038/s41467-019-09959-4, published online 13 August 2019.

The original versions of Fig. 1, Fig. 2 and Fig. 6 in this Article have been updated.

Figure 1b, c have been updated to remove an individual’s name and associated data.

Figure 2c has been updated to anonymize the data.

Figure 6 has been updated to remove individuals’ names associated with the light-blue data points in the top-right.

This has been updated in both the PDF and HTML versions of the Article.

