# Addendum: Discrepancy in scientific authority and media visibility of climate change scientists and contrarians

**DOI:** 10.1038/s41467-020-14507-6

**Published:** 2020-01-23

**Authors:** Alexander Michael Petersen, Emmanuel M. Vincent, Anthony LeRoy Westerling

**Affiliations:** 1 Management of Complex Systems Department, Ernest and Julio Gallo Management Program, School of Engineering, University of California, Merced, CA 95343 USA; 2 Medialab, Sciences Po, 75007 Paris, France; 3 Center for Climate Communication, University of California, Merced, CA 95343 USA; 4 Sierra Nevada Research Institute, University of California, Merced, CA 95343 USA

**Keywords:** Computational science, History, Climate change

Addendum to: *Nature Communications* 10.1038/s41467-019-09959-4, published online 13 August 2019.

With this addendum, we wish to clarify objectives, operational choices, and related assumptions made in the selection and analysis of two sets of individuals based upon publicly available noninterventional source data.

The motivation for our study is to provide insights into sociotechnical dimensions of climate change (CC) communication in a way that shows how hybrid print and digital media have contributed to the broad production and dissemination of information that runs counter to available evidence concerning trends, attribution, impact and scientific consensus levels relating to CC.

Against this background, we performed a data-driven analysis juxtaposing equal numbers of individuals associated with the consensus and nonconsensus viewpoints. Individuals were separated into two groups based upon the primary data sources listed below, from which we extracted the full names of all associated individuals. Then, for each individual identified, we applied the same data collection and analysis methods to measure both their media visibility and scientific productivity and citation impact pertaining to CC science.

## Climate change contrarian (CCC) definition and data source

We defined climate change contrarians (CCC) as those that appeared on these three open web-based sources:

1. The Heartland.org conference website: http://climateconferences.heartland.org/speakers/
2. The Nongovernmental International Panel on Climate Change (NIPCC) website: http://climatechangereconsidered.org
3. The DeSmog website: https://www.desmogblog.com/global-warming-denier-database

Following similar terminology in the literature, such as climate change countermovement^1–3^, we chose the term “contrarian” as a person that takes up a contrary position that is opposed to that of the majority. This term is not negatively charged and thus is the best match for our study scope^4^. Individuals for which this definition applies derive from a wide range of backgrounds, including politics and business, in addition to practicing academics and scientists. In particular, this definition does not depend on or infer a given individual’s expertize, political opinion, or other defining characteristic other than described here.

We used this definition because the majority of individuals named in these sources have demonstrably expressed and disseminated contrarian views outside of traditional scientific discourse and/or have been relied upon by either contrarian organizations or the media to represent contrarian views; here, we define contrarian views to be nonconsensus views that oppose what most scientists consider to be established facts—that climate change is currently occurring, that humans are primarily responsible, that the scale of the change and its impacts is significant, and that there is nearly full consensus within the scientific community in this regard.

We used those sources to collate a list of 386 individuals:

Individuals who have authored, signed, or otherwise contributed to the Heartland conference and related NIPCC documents (see list above) yield 226 name identifiers; the remaining 160 individuals are sourced from Desmogblog, and include individuals who are engaged with contrarian messages and organizations to varying degrees.

## Climate Change Scientist (CCS) definition and data source

In assessing authority in climate change science we based our selection on total impact (number of citations) in journals included in the Web of Science (WoS). We made this operational choice because peer-reviewed journal article publications and citations are the commonly used standard of academic productivity and impact—and the accepted journals of record on climate change science are indexed by WoS. We assume that individuals claiming a high level of authority on climate change science would be expected to have some footprint within the corpus of articles on climate change indexed by WoS.

We looked only at articles which met a broad search criteria identified by our WoS front-end search portal query by “Topic” = (Climate NEAR/5 Change OR Global NEAR/5 Warming OR Climatology OR Climate NEAR/5 Model OR Climate NEAR/5 Extreme) AND (Climate). This reproducible search query identifies roughly 200,000 research articles from various research streams contributing to the CC literature, while reducing the inclusion of related research streams that address climate but not necessarily climate change. Consequently, a scholar could be quite accomplished in another distant or even proximal field such as meteorology, with numerous publications and citations, and these would not count in our assessment.

As such, we selected the 386 researchers (denoted by CCS) with the largest citation totals across their CC publications as representatives of scientific authority on CC. Among these 386, we identified eight individuals who were also externally identified within the CCC group as the result of one of two mechanisms:

1. author credit misattribution deriving from the author name ambiguity problem, which we addressed extensively in the methods section of our original article; in other words, this is to say that several of these eight individuals had common last surnames which erroneously mapped onto another prolific climate scientist(s), and so their inclusion in the CCS group corresponds to a false positive error;
2. a few individuals have made considerable contributions to CC research, but have nevertheless associated with contrarian views (supporting evidence within the three sources listed above).

Indeed, an overlap in the two groups of this magnitude (8/386 = 0.0207) is rather consistent with what one could expect to find in our sample according to surveys on consensus among scientists^5,6^. In order to remain objective, we opted not to modify the externally validated sets of individuals, and so these eight individuals were summarily kept within the CCC group.

Consequently, the contrarian group does include a few individuals who have previously contributed to highly-cited climate science research. Adjusting for this ~1% overlap between groups would not alter our study’s main findings.

## Media data and data analysis

Media article metadata (including article title, publication date, media source and web hyperlink—but not full article text) were obtained from the public Media Cloud project (https://mediacloud.org) using their open web-based API to query and retrieve source data^7^.

We operationalized the comparison of CCC and CCS by simultaneously measuring how frequently contrarians are cited in the media, and how authority in peer-reviewed scientific discourse aligns with media visibility, in particular accounting for media source type. As illustrated in Fig. 4 of our original analysis: even conditioning individuals on a particular level of scientific authority (based upon productivity or citation metrics), we found that CCC exhibit a disproportionate media visibility when compared with CCS of similar authority level; at the group level, we show that the distribution of the ratios (*r*_*p*_ and *r*_*c*_), measuring the media visibility normalized by either scientific productivity or citations, respectively, show a significantly broader distribution for CCC as compared with CCS.

Media visibility is here defined as the frequency with which an individual’s name is mentioned in the media. There is no positive or negative connotation to this visibility, as the context of such mention is not investigated or taken into account.

Our analysis does not aim to measure the effect of being a scientific contrarian on media visibility. Nor are we simply seeking to demonstrate that these selected contrarians have higher media visibility than their career scientist counterparts; similarly, we are not seeking to demonstrate that career scientists have higher levels of authority in science—both of which would be tautological given our group selection criteria. Instead, our results are based upon the simultaneous juxtaposition of differences in prominence across two distinct communication channels—media and scientific publication.

## Study limitations and uncertainties

One core assumption is that the 386 CCC analyzed in our study represents the most important sources of nonconsensus views on CC. We are confident that the CCC group captures a relatively comprehensive snapshot of individuals regarded as contrarians, albeit with varying degrees of misalignment with the scientific consensus. At the same time, excluding any of them would not qualitatively change the results of this analysis since our data are comprehensive at both the unit of analysis and unit of observation level, and our study’s statistical results are demonstrably robust at both the individual and group levels.

There are, however, significant differences in group composition characteristics. In particular, there is considerable variation in professional background and degrees of nonconsensus positions across individuals belonging to the CCC that is not inherent among the CCS. This compositional difference is the most significant limitation to performing a counterfactual comparison between consensus and nonconsensus scientific actors—as the population size of the latter group among practicing academics and scientists is only around a few percent according to several surveys^5,6^. Moreover, nonconsensus views can change over time, e.g., as new evidence arises that causes an individual’s perspective to shift. There are of course other sources of positional change, as highlighted by research showing that the communication strategy for dissent within contrarian organizations is shifting from trend and attribution skepticism to more subtle forms^8^, which would further complicate an analysis oriented around article-specific context pertaining to individuals’ media visibility.

Thus, we focused our analysis on simple and objective counts of publicly available information, using new data collection tools leveraging the intersection of Media Cloud and WoS data, that have not previously been applied to this arena. Based on our multilevel approach (performed at both the group and individual levels of comparison), the overall message of the study remain unchanged, despite the marginal levels of classification error and the balanced sources of misattribution error, as discussed here as well as in the original manuscript, which give rise to a few individuals in the CCC group who also classify as highly cited scientists in climate change research.
