# Supplementary material for: Discrepancy in scientific authority and media visibility of climate change scientists and contrarians
Source: Nat Commun. 2019 Aug 13;10:3502. doi: 10.1038/s41467-019-09959-4 (PMC6692310; doi:10.1038/s41467-019-09959-4)
Supplement: Supplementary file 1 — Supplementary Information [file 41467_2019_9959_MOESM1_ESM.pdf]

Supplementary Information:

Discrepancy in scientific authority and media visibility of climate change scientists and contrarians

Alexander Michael Petersen [1]

Emmanuel M. Vincent [2,3]

Anthony LeRoy Westerling [1,3,4]

[1] Management of Complex Systems Department, School of Engineering, University of California, Merced, CA 95343

[2] Medialab, Sciences Po, Paris, 75007 France

[3] Center for Climate Communication, University of California, Merced, CA 95343

[4] Sierra Nevada Research Institute, University of California, Merced, CA 95343

Please send correspondence to: AMP (apetersen3@ucmerced.edu) or EMV (mel.manux@gmail.com) or ALW (awesterling@ucmerced.edu)

Supplementary Figures S1-S6

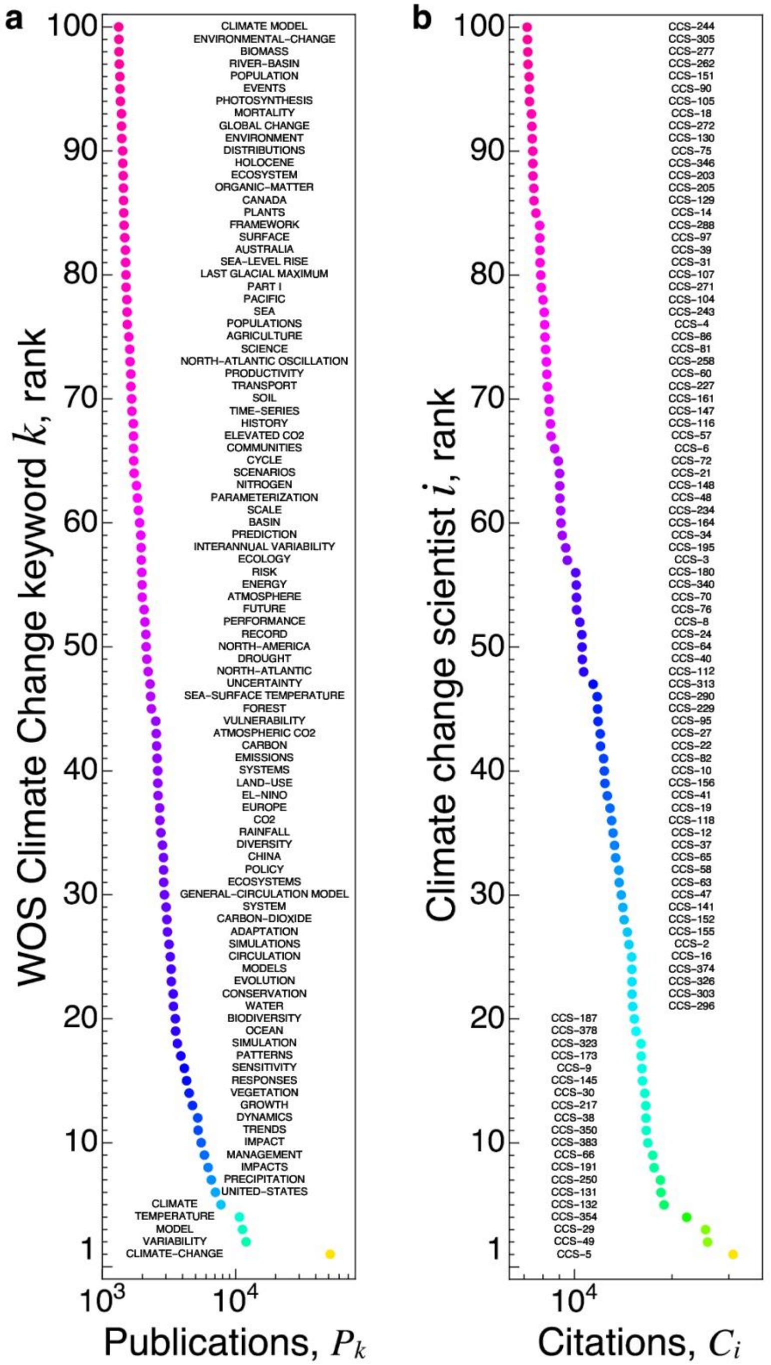

Supplementary Figure 1. **Topicality and highly-cited researchers within the CC corpus comprised of 0.2 million publications indexed by the WOS.** (a) List of the top-100 Web of Science controlled vocabulary keywords ranked according to the number of publications in our WOS sample including a given keyword; not surprisingly, Climate-Change is the most prominent keyword, associated with roughly 1 out of every 3 publications. (b) List of the top-100 researchers on climate change, ranked according to their total number of citations, C_i.

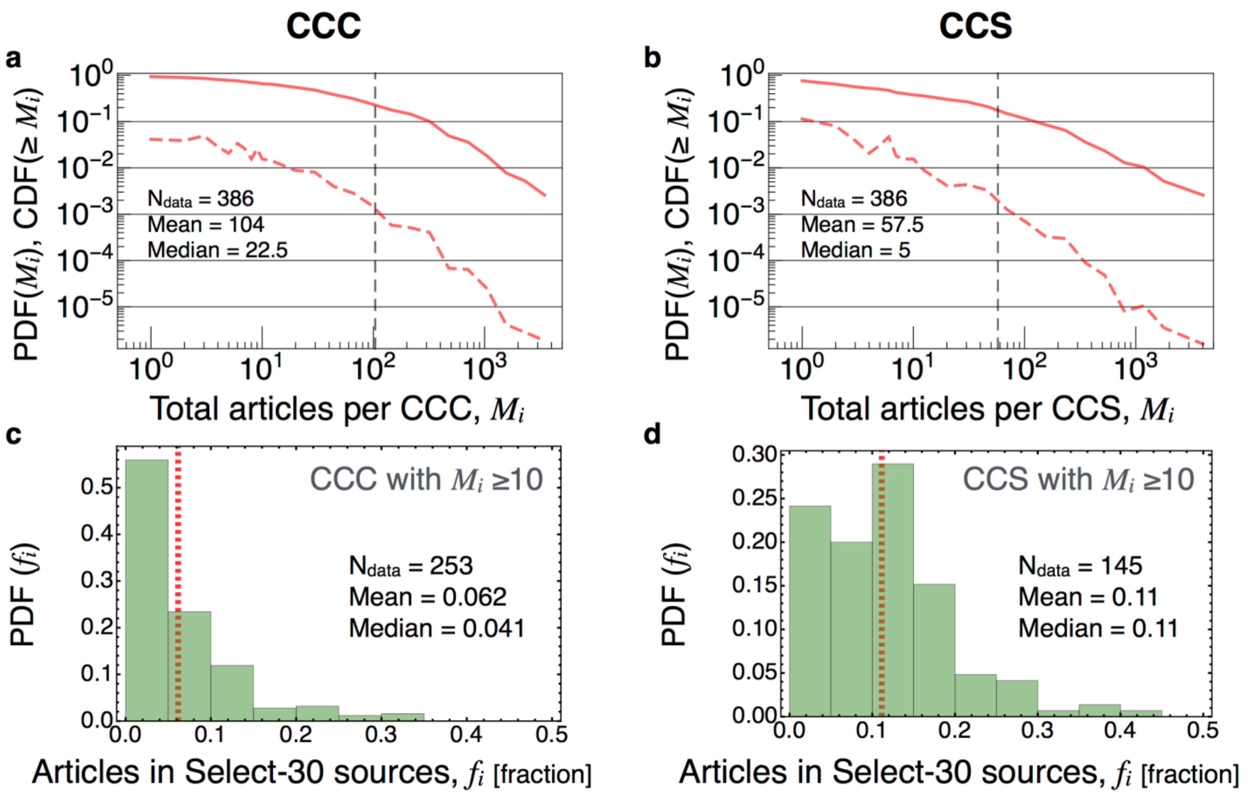

Supplementary Figure 2. **Distribution of media activity by individual.** Probability distributions by group: (a,c) CCC; (b,d) CCS. (a,b) Right-skewed distribution of the number of media articles M_i associated with individual i; vertical line indicates the distribution mean. (c,d) Distribution of the fraction f_i of articles in select-30 media sources, computed using just individuals with sufficient prominence (M_i≥10 articles); vertical line indicates the distribution mean.

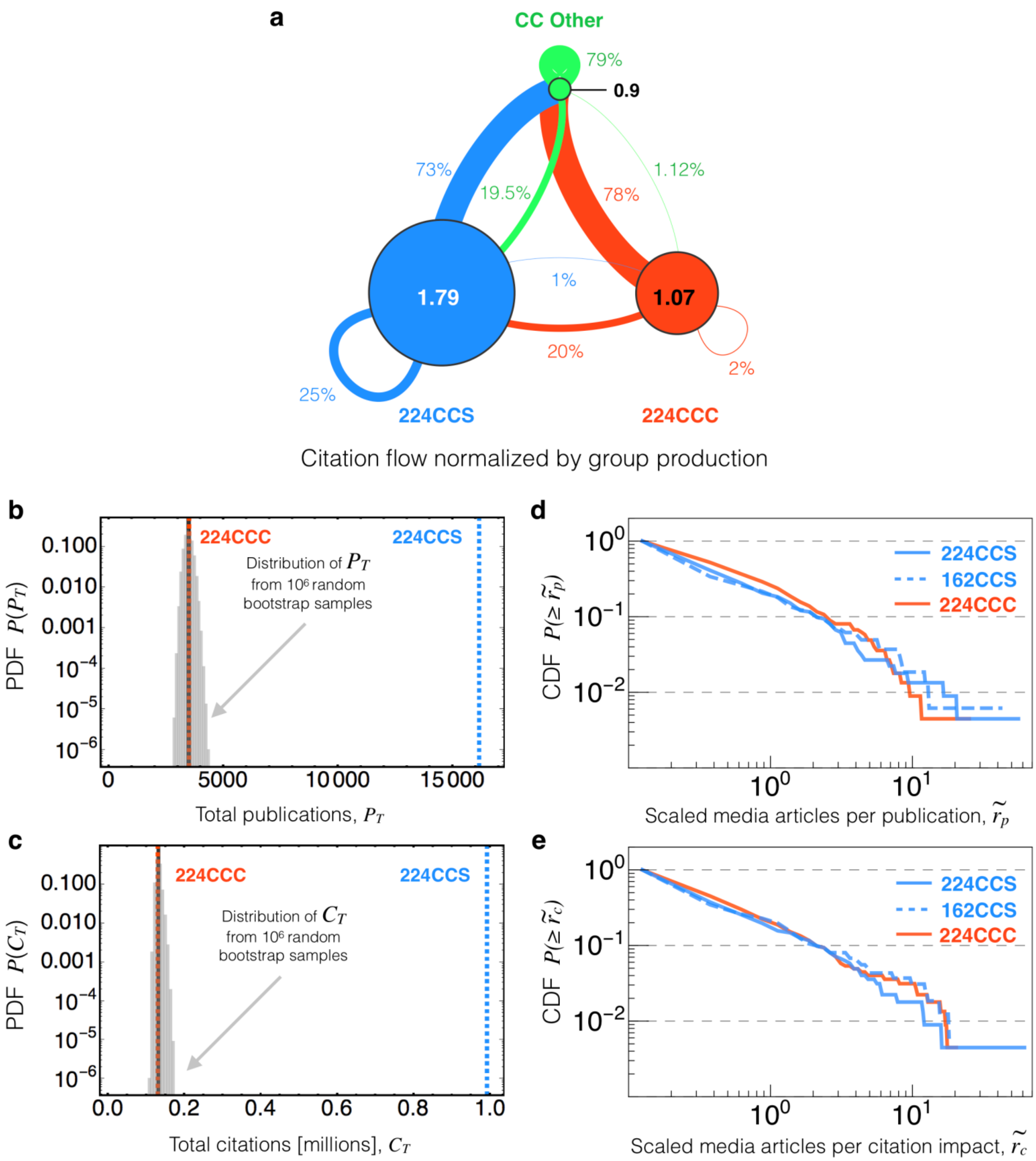

Supplementary Figure 3. **Climate Change authority – group level.** (a) Citation flow between individuals belonging to three groups: CCC, CCS, and CC Other. Citations between groups normalized by the quantity produced by a given group (i.e. each group of links of the same color add to 100%); e.g. 1% of the citations produced by CCS are directed towards the CCC, whereas 20% of the citations produced by CCC are directed at the CCS group. Node size represents the ratio of incoming citations received divided by the citations produced by a given group, e.g. CCS receives 1.79 times as many citations as it produces. (b,c) Demonstration of the significant disparity in scientific authority between the CCC and CCS groups as a whole. (b) Random

sampling distribution for the total number of publications by the 224 members of each CC group: the distribution mean is indicated by the vertical black line, and the real totals for CCC and CCS correspond to the red and blue dashed lines. (c) Random sampling distribution for total number of citations: the distribution mean is indicated by the vertical black line, and the real totals for CCC and CCS correspond to the red and blue dashed lines. (d) Cumulative distribution P($\geq \tilde{r}_p$) of media visibility per publication, $r_{p,i} \equiv M_i/P_i$.In order to demonstrate that the three distributions are qualitatively similar across the three datasets shown, we calculated the cumulative distribution using scaled values $\tilde{r}_{p,i} \equiv r_{p,i}/\langle r_p \rangle$, whereby individual $\tilde{r}_{p,i}$ values are normalized by the group mean value $\langle r_p \rangle$. In addition to the distributions calculated for the 224CCS and 224CCC groups, the dashed blue CDF corresponds to the distribution calculated for the remainder of the CCS group (162CCS). The mean value $\langle r_p \rangle$ for each group is: 15.4 (224CCC), 1.04 (224CCS), 1.66 (162CCS). (e) Cumulative distribution P($\geq \tilde{r}_c$) of the scaled visibility per citation impact, $\tilde{r}_{c,i} \equiv r_{c,i}/\langle r_c \rangle$. The mean value $\langle r_c \rangle$ for each group is: 18.8 (224CCC), 0.94 (224CCS), 0.47 (162CCS).

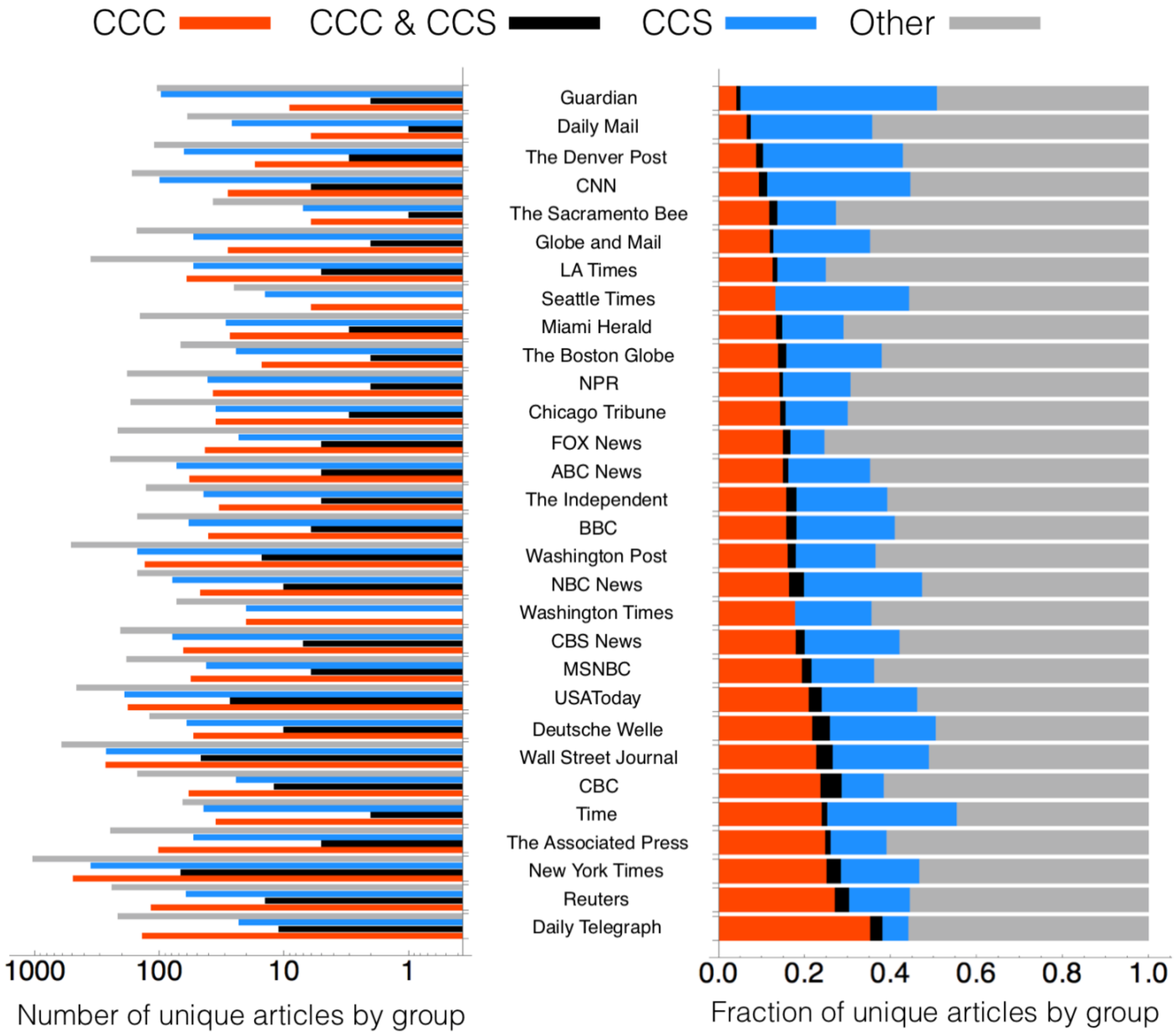

Supplementary Figure 4. **Coverage of CCC and CCS by the 30 select Media Sources.** Unique articles by each media source categorized by the total number and fraction that feature just CCC (red), just CCS (blue), both CCC and CCS (black), and other CC topics and authorities (grey). We found 11,233 unique articles from these select-30 media sources, which corresponds to 11% of the total number of unique articles analyzed (102250). Total number (%) of articles by group: CCC = 2203 (20%), CCC&CCS = 283 (2.5%), CCS = 2180 (19%), Other = 6567 (58%).

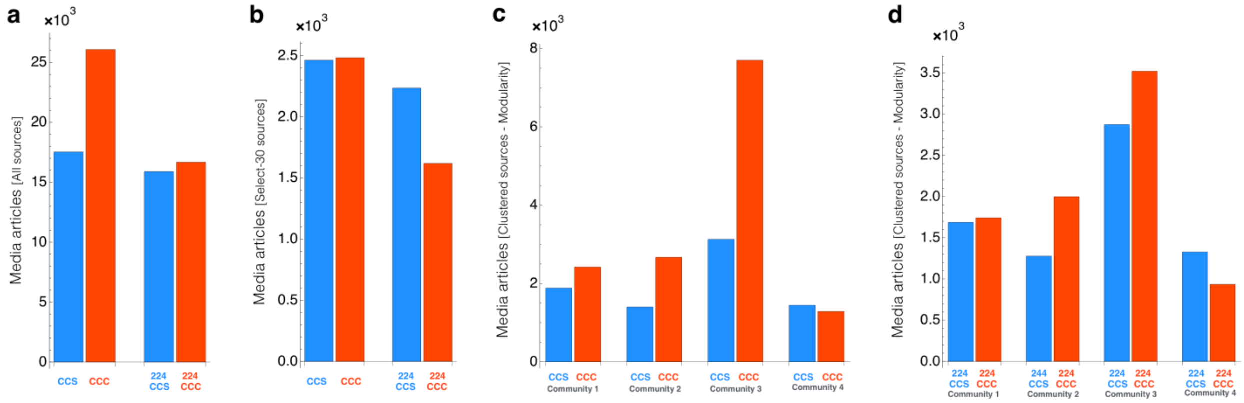

Supplementary Figure 5. **Media Cloud article counts – permutations of CC group size and Media Source groups.** (a) All media sources: comparison of tallies for the complete and refined groups – of size 386 and 224 individuals, respectively. 224CCC indicates the subset of 224 CCC comprised of just the individuals with at least one WOS publication; 224CCS indicates the 224 most-cited CCS. (b) Only select-30 media sources: comparison of tallies for the groups of size 386 and 224. (c) All media sources, clustered into 4 communities produced by the Louvain clustering algorithm [48]: comparison of tallies for the complete groups of size 386. (d) Similar to (c) but tallies are calculated for just the groups of size 224.

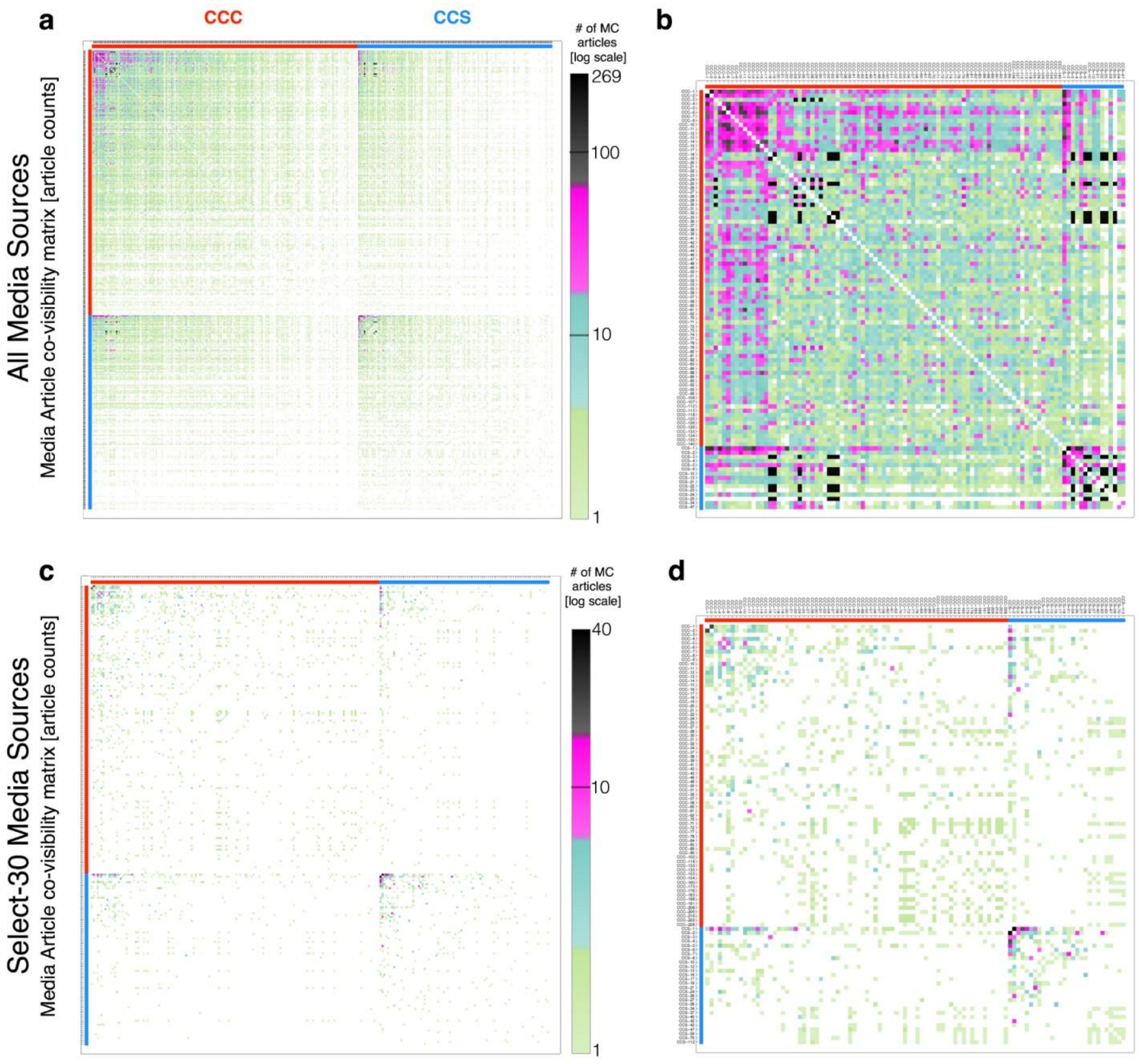

Supplementary Figure 6. **Co-visibility in media articles.** Each panel shows the (symmetric) matrix elements M_{ij} representing the number of MC articles featuring both individual i and j across the set of media sources indicated. The individuals are ordered within their respective groups according to their total visibility across all media sources, with the CCC grouped first (indicated by red outline; names are anonymized to foster privacy) and the CCS second (indicated by blue outline). Most co-visibility is within each CC group, and so by construction, the upper right and bottom left quadrants of panels (a,b) are more empty than their respective upper left and bottom right quadrants. Panels (c,d) provide a magnification of the regions with high co-visibility. We applied a modularity maximizing algorithm to cluster individuals into groups [48], which identified three roughly equal-sized groups: 2 mixed communities and one extremely polarized echo chamber community comprised primarily of CCC; see Figure 6 for the clustered network layout representation of the co-visibility matrix. Each color scale shows the range of $Log_{10}\, M_{ij}$ values, partitioned into quartiles; white cells indicate $M_{ij} = 0$. (a) M_{ij} calculated using all media sources, showing all individuals with at least one shared appearance

(i.e. an individual from CCC or CCS does not appear in the matrix if he/she does not appear in an MC article with at least one other individual CCC or CCS). (b) The matrix of the 100 most active individuals from panel (a). (c) M_{ij} calculated using only the select-30 mainstream media sources, showing all individuals with at least one shared appearance. (d) The matrix of the 100 most active agents from panel (c). There is a disproportionate representation of CCC since they have a higher tendency to be associated with the same MC article. By panel, the number of rows (and percent of total in parentheses) represented by each group are: (a) CCC = 357 (58%) and CCS = 261 (42%); (b) CCC = 85 and CCS = 15; (c) CCC = 150 (63%) and CCS = 88 (37%); (d) CCC = 72 and CCS = 28.

Supplementary Table S1

Media Source name	First issue date	Media type	MC unique identifier	# Unique MC articles (CCC+CCS)	# Unique MC articles (All)
Guardian	1821	Daily newspaper (broadsheet)	1751	910	1949
New York Times	1851	Daily newspaper (broadsheet)	1	583	1188
Washington Post	1877	Daily newspaper (broadsheet)	2	395	854
Daily Mail	1896	Daily newspaper (tabloid)	1747	295	806
Reuters	1851	International news cable (radio, print, tv, internet)	4442	118	473
FOX News	1996	U.S. news cable (tv, internet)	1092	192	431
Daily Telegraph	1855	Daily newspaper (broadsheet)	1750	159	406
Washington Times	1982	Daily newspaper (broadsheet)	101	170	387
The Sacramento Bee	1857	Daily newspaper (broadsheet)	26	134	380
MSNBC	1996	U.S. news cable (tv, internet)	1149	149	354
The Associated Press	1846	International news cable (radio, print, tv, internet)	1154	133	298
Time	1923	Weekly magazine	4419	71	287
LA Times	1881	Daily newspaper (broadsheet)	6	104	287
USA Today	1982	Daily newspaper (broadsheet)	64866	135	285
The Independent	1986	Daily newspaper (until 2016, now exclusively web-based)	23132	80	260
The Denver Post	1892	Daily newspaper (broadsheet)	31	104	253
Wall Street Journal	1889	Daily newspaper (broadsheet)	1150	94	244
Miami Herald	1903	Daily newspaper (broadsheet)	28	73	243
BBC	1922	British news cable (radio, tv, internet)	1094	123	243
ABC News	1945	U.S. news cable (radio, tv, internet)	39000	83	235
CBC	1941	Canadian news cable (radio, tv, internet)	7333	108	212
The Boston Globe	1872	Daily newspaper (broadsheet)	15	82	209
CNN	1980	U.S. news cable (tv, internet)	1095	58	202
CBS News	1927	U.S. news cable (radio, tv, internet)	1752	83	193
NPR	1971	U.S. news cable (radio, internet)	1096	81	146
Chicago Tribune	1847	Daily newspaper (broadsheet)	9	40	112
Globe and Mail	1844	Daily newspaper (broadsheet)	19477	41	108
Deutsche Welle	1953	German multi-language news cable (radio, tv, internet)	1708	33	92
NBC News	1940	U.S. news cable (radio, tv, internet)	25499	14	51
Seattle Times	1891	Daily newspaper (broadsheet)	24940	20	45

Supplementary Table 1. **List of select-30 mainstream media sources.** Unique MC articles refers to the total number of articles by a given media source in our dataset after merging different articles with the same title. The fifth column counts only the articles featuring CCC and CCS, whereas the final column reports the number of unique articles across all 102,250 articles collected from MC on CC. See Supplementary Figure 4 for the coverage of CCC and CCS by each media source.

Supplementary Notes 1 & 2

Supplementary Note 1

Identification of prominent CCC and CCS: We collected the names of CCC from The Heartland Institute (a central climate change contrarian/denial organization that produces pseudo-scientific reports for distribution to the public [63], which have been subsequently debunked by prominent and active CC scientists), and the website of the DeSmogblog project (which hosts profiles documenting the activities of 300 prominent contrarians). We then completed this list with 16 additional signatory authors of the most recent NIPCC report who were not included in either of the first two lists, resulting in a total of 386 CCC individuals.

We obtained a list of prominent CCS by ranking the researchers within the WOS dataset according to their total citation impact. To be specific, for each publication p we recorded c_p, the number of citations received by a given p through April 2017 according to WOS. Because we are concerned with obtaining a list of the most prominent researchers on CC, we are not concerned with the measurement bias associated with combining citation counts for recent publications with those for older publications - in essence, the list of highly-cited researchers should not be dramatically impacted by this right-censoring statistical bias because the CC domain is old enough that the prominent leaders of the community have long been established.

Author name disambiguation strategy: Our analysis of CC researchers is challenged by the classic author name disambiguation problem because WOS does not uniformly record the full first names of the authors in all of their historical records. Thus, one of the main limitations in using the Web of Science data is that we only have the first name initial to distinguish authors with the same last name.

In order to reduce the impact of such false attribution – resulting in a type-1 false-positive error by which publications from different authors with similar abbreviated names are falsely clumped together – we implement a rare surname disambiguation method which has been shown to work remarkably well given its straightforward approach [45]. One reason why this method is well-suited for our present context is because achievement statistics in science are extremely right-skewed (or heavy-tailed), meaning that the net citation tally of an eminent scientist can easily exceed the average scientist within a particular domain by a factor of 100 or more [44, 46]. Thus, this method is particularly well-suited for the case of studying the cumulative citation tallies of top achievers, since it can be assumed that the percent error due to clumping for the most prominent scientists is relatively small due to their extreme citation tallies.

The basic assumption behind the rare surname approach is that there is a higher likelihood that two or more articles that feature this same surname and first name initial in fact correspond to just a single researcher – in other words, it is assumed that the probability of false-positive clumping error is smaller when the surname is less common. Along these lines, manual inspection confirms that most of the CCS and CCC have relatively unique surnames, and so this method is amenable in this regard.

We initialize the unique name disambiguation method by first tallying the first initials in the dataset for each surname; from this first pass we obtained a list of 202,152 unique first initial + surname combinations, e.g. in the case of common surnames such as Smith or Jones we keep track of the variants we encounter such as A. Smith, B. Smith, C. Smith, etc. For a given surname, we then count the number of variant types based upon the number of unique first initials. By way of extreme example, the most common surnames, e.g. Wang and Lee, have the maximum 26 variant types, one combination for each standard letter of the new Latin alphabet.

From this master list of surname and first initial combinations we can assess the degree to which a surname is rare. We then proceed to refine the master list of unique first initial + surname combinations by excluding all surnames that have more than a single type of first initial. The results of this pruning was to cut the list down to 96,475 first initial + surname combinations.

Nevertheless, such a strict criteria excludes a significant number of the CCC as well as prominent CCS with common last names. Thus, in order to compromise between a list of high-confidence disambiguated surnames and a list which excludes a large number of true prominent CC researchers, we created a separate list of common surnames up to first-name initial degeneracy of 21. We then compared the top-1000 cited lists obtained using the two extremes: first, using all surname combinations (degeneracy = 1); and second, using degeneracy 21. We identified 710 overlapping first initial + surname combinations in these two lists, and added these names to the master list of 96,475 unique surnames. In order to assure that all 386 CCC were included in this master list, we performed on final step which was to add any of their first initial + surname combinations that were not on the list. The final result after these steps was a list of 97,398 first initial + surname combinations for the final dataset.

Supplementary Figure 1b shows the 100 most-cited CCS, ranked according to the citation tally C_i calculated by taking the linear sum $C_i = \sum^{i \in p} c_p$ across the set of papers corresponding to a given researcher name, indexed here by i. Again, since we are mainly concerned with identifying a comparable set of 386 prominent CCS, we are not concerned with accounting for publication team size, author order, or other credit attribution factors. Instead we opt for a straightforward definition for the citation impact measure C_i. We also noted that seven top-cited researcher profiles belonging to the CCC list. These individuals were summarily kept within the CCC group, and their places within the 386 CCS list were replaced with the next highest-cited researcher profile. It is instructive to consider the net total in terms of two contributions, $C_i = C_i^{\text{true}} + C_i^{\text{misattribution}}$, the total citations from the author's true set of publications and those that are misattributed to him/her, respectively. To illustrate how less-prominent researchers (i.e. CCC) benefit more than prominent researchers from publication misattribution, consider first that prolific career scientists have C_i^{true} in the range of 10^3 to 10^5 WOS citations [44], which are orders of magnitude greater than the citations accrued by the average papers in their field. It then follows that $C_i^{\text{true}} \gg C_i^{\text{misattribution}}$. Thus, the type-1 error corresponding to the false attribution of papers to the publication portfolios of CCS only marginally increases their true citation tallies. Conversely, clumping of two or more less-cited researchers together can significantly boost the net citation tally of the less-prominent CCC, many of which are politicians and business-people who have no scientific publication record but share the same name with active CC researchers, e.g. S. BANNON. Thus, profiles of this type are characterized by $C_i^{\text{true}} < C_i^{\text{misattribution}}$. Indeed, there are many similar CCC who have not published a single scientific article on CC yet who contributes to the CCC totals through misattribution. We

checked to see how many CCC profiles had at least one article in the WOS dataset and found that only 224 CCC had at least 1 publication within our WOS dataset. Thus, in an effort to make the comparison more fair, we provide additional comparisons using just the subset of 224 published CCC (denoted by 224CCC) with the 224 most-cited CCS (denoted by 224CCS) in Figure 3, Figure 7, Supplementary Figure 3 and Supplementary Figure 5.

The asymmetric network of scientific CC authority: In order to estimate the attribution of scientific authority between the two CC groups in a self-consistent manner, we analyzed the network of citations between the CCS, CCC, and a third set comprised of roughly ~50,000 other CC scientists (denoted by CC Other). This latter group is comprised of only those authors who are connected within the citation network, which is roughly half of the total 96,475 researcher profiles identified in the previous section. The other ~40,000 researchers are infrequent authors who likely appear on just a single article which did not cite nor was cited by any other article in the dataset, and for this reason they are not connected within the citation network.

Citations in the WOS dataset represent a directed attribution between two peer-reviewed scientific articles, one citing and one cited. These citations can be classified as positive, i.e. researchers attributing and building on prior research, as well as negative, consisting of pointed disagreement or critique of prior research methods or interpretations. Regarding the frequency of negative citations in science, a recent study on citation patterns in the *Journal of Immunology* found that 2.4% of the total citations were of the negative type, with 7.1% of the publications in their study receiving at least one negative citation [50]. It remains to be shown to what degree these rates of negative or critical citation are discipline dependent, but it is likely that they are higher in more politically controversial fields, especially those in which there are diametrically opposed groups around contentious issues [51].

The consensus on the anthropogenic roots of climate change among active researchers has been demonstrably solid since the early 2000s [1-4]. More recently, a large 2013 study of nearly 12,000 abstracts of papers published between 1991-2011 reports that among the 1 in 3 abstracts stating a definite position on the anthropogenic issue, 97% of these positions *endorse* anthropogenic (human) factors causing global climate change [3]. In other words, the volume of research rejecting anthropogenic global warming has been and continues to be a very small fraction of the total research on the topic over the study period.

It is far beyond the scope of our analysis to perform a content analysis on the nature of positive and negative citations between the CCC and CCS. We can, however, infer differences in scientific authority from the variations in the patterns of citation flow between the CCC and CCS, while also using the CC Other as a third comparison set.

To this end, we used the unique digital object identifier (DOI) associated with each CC publication to keep track of how many times each publication was cited by other publications within our CC dataset. We then constructed the group-level citation networks shown in Figure 7a and Supplementary Figure 3a, as well as individual-level citation networks shown in Figure 7b. Of the 224 CCC who have coauthored at least one article on CC in our WOS dataset, only 168 are connected within, and thus contribute to, the group-level and individual-level citation networks; 223 of the 224 matched CCS are connected within and contribute to the intra-CC citation network.

Figure 7a shows the citation flow aggregated at the group level, including the CC Other group comprised of the remaining 50,051 researchers within the CC citation network. This group-level network shows the levels of co-citation between groups as a percent of the total citations produced – while both groups of 224 represent 0.44% of the total researchers analyzed, only 1.1% of the citations within the network are directed towards the CCC whereas 20.2% percent are directed towards the CCS. Using the CC Other as an external source of citation credit production, we calculate that 17 times as many citations flow from the CC Other to CCS as compared to CC Other to CCC.

This citation disparity is partially due to the different publication levels across groups. Thus, Supplementary Figure 3a shows the citation flow normalized by the total outgoing citations produced by each group, to account for the fact that the CCC produce fewer publications, and thus fewer citations, than the CCS. For example, the link weights between CC Other and CCS and CC Other and CCC still have the ratio 19.5/1.12 = 17. However, when controlling for each group's productivity, the scientific credit attributed to the CCC by the CCS and CC Other is again only 1% of what is produced by each of these two groups individually. Moreover, this normalizing by group productivity reveals that the CCS receive 79% more citations than they produce, a consistency check confirming their authority on CC science.

Thus, both estimations of citation flow show that the CCS group has roughly 20 times the scientific impact as the CCS, which is a lower bound estimate due to the name disambiguation problem described in the previous section. In the next section we estimate the significance level of this disparity.

Estimating the significance level for the disparity in scientific Climate Change authority: Is the factor of 20 difference in scientific impact between the CCC and CCS groups large or small? To address this question we performed a bootstrapping estimation by randomly sampling from the empirical distributions of productivity (of individual researchers) and citations (of individual publications). As such, this random sampling facilitates estimating the statistical significance of the difference between the CCC and CCS groups. Moreover, this analysis also provides an estimation of how the scientific achievements of the CCC group would compare to more modest groups of CC scientists.

We first consider the total number P_T of publications produced by each group, leading to the natural question – how significant is the difference between $P_T = 3{,}511$ for the CCC and $P_T = 16{,}167$ for the CCS? Here we calculate $P_T = \sum_{i=1}^{224} P_i$ as the sum over the individual publication tallies P_i for members of each group. Because this method does not account for multiple authorship of the same publication, the P_T calculated for each group is slightly larger than the total number of *unique* publications per group reported in Figure 3b. Calculating P_T as a sum over individuals permits random sampling of individuals from the observed productivity distribution, which is more straightforward to implement.

We estimated the likelihood of obtaining a given P_T value by randomly sampling 224 draws (without replacement) from the 5,314 researcher profiles with $P_i \geq p_x$ publications (not including the 224 CCC and the 224 most-cited CCS). We then counted the total number of publications for each random sample, and repeated this sampling 10^6 times. Supplementary Figure 3b shows the probability distribution $P(P_T)$ of the 10^6 bootstrap samples along with the

empirical P_T values for both the CCC and CCS groups. We use $p_x \equiv 8$ so that the average random sample value $\overline{P}_T = 3{,}482$ most closely matches the real $P_T = 3{,}511$ value for the CCC; also, we observe that 41% of the random groups have $P_T > 3{,}511$, further demonstrating the goodness-of-fit for this particular threshold p_x. More importantly, we observe no random P_T values that are even close to the $P_T = 16{,}167$ value for the CCS group (the maximum random P_T value observed was 4,406), thus demonstrating the significance of the productivity gap between CCS and CCC groups.

Likewise, we performed analogous sampling without replacement of individual publications in order to estimate the statistical significance of the difference in the total citations between the two groups. To be specific, we estimated the CCC group citation impact C_T by randomly selecting 3,367 publications and tallying their citations. Importantly, we also conserved the distribution of publications years in our sampling procedure so that each random sample matches exactly the real distribution of publication years. For example, we recorded 62 articles published by CCC in 2000, thus we randomly selected this same amount from the set of all articles published in 2000, repeating the procedure for each year in which the CCC have at least a single publication.

In order to meet this publication-year-conserving sampling condition while also best matching the empirical value $C_T = 130{,}833$ citations for the CCC, we provided each random publication draw a handicap amounting to a minimum of $c_x = 6$ citations. That is, if we drew a publication with less than c_x citations, we artificially inflated the count up to c_x citations. This rule was necessary to overcome the large fraction of articles that are cited just a few times, a generic property of citation distributions; we choose this c_x value so that the random distribution average best matched $C_T = 130{,}833$ (mean value $\overline{C}_T = 132{,}446$, with 58% of the random samples having $C_T > 130{,}833$ and maximum random total equal to 178,320). We performed this random sampling 10^6 times. Supplementary Figure 3c shows the probability distribution $P(C_T)$ for random citation totals along with the empirical C_T values for the CCS and CCC groups. As in the case of productivity, we show that there is an infinitesimally small likelihood of obtaining such a large difference by chance – thereby demonstrating the statistical significance and magnitude of the citation authority gap between the CCC and CCS.

In summary, we compared the group-level authority of CCC and CCS deriving from publication in scientific journals that meet the WOS rigorous indexing standards. The productivity and citation impact differences between the CCC and CCS groups are unlikely to arise just due to chance; rather, the group-level publication and citation totals for the CCC group are statistically indistinguishable from a random sampling of relatively baseline research profiles – far removed from the career achievements of elite CCS.

Supplementary Note 2

CCC and CCS in the media: We obtained a list of M_i media articles associated with each individual i by querying the MC database using the full name of the individual + the string *climate* over the time period 01/01/2000-05/01/2017. We use the modifier *climate* instead of *global* because the latter could trigger articles from the MC database having to do with global affairs that are not necessarily related to CC. 2005 saw the emergence of articles associated with the search term *Climate Skeptic*, which yields a factor of 10 fewer articles than the combined search term *Climate Change Global Warming*. Together, we downloaded 121,729 unique articles produced by 7,126 unique media sources (30,934 unique MC articles for the CCC; 22,592 for the CCS; and the remainder for the blanket query *Climate Change Global Warming*). We then applied an article disambiguation to merge articles with similar title from the same media source, but with different URL and MC unique identifier. This refinement, detailed in the Methods section, reduced the total dataset size to 102,250 MC articles, corresponding to a 16% reduction.

Importantly, these numbers do not include media articles published after 10/01/2016, which we excluded in order to avoid auxiliary media articles relating to CC that had more to do with the 2016 US Presidential election and the subsequent selection of administration appointments.

Figure 2 shows the number of MC articles for the most prominent individuals (indexed by i) and media sources (indexed by s) from each of the CCC and CCS groups. Figure 3 shows the group-level counts. However, since MC includes articles from a wide range of media sources, including second-tier news sources and even personal blogs, we calculated the group-level article totals using three methods: first, calculating across all media sources; second, calculating across a select subset of 30 mainstream media sources listed in Supplementary Table 1; and finally, calculating tallies across subsets of media sources defined by an unsupervised clustering algorithm.

Group-level comparison based on these three complementary methods facilitates accounting for the wide variation in media source size, age, and quality. The results of the first counting method captures the total visibility of each group across all media sources. The results of the second counting method captures their visibility in longstanding and widely-trusted media sources. And the results of the third counting method captures their visibility in subsets of media sources that tend to include the same CCC and CCS. Importantly, method (c) relies on a data-driven method to group media sources, as opposed to method (b) which uses a select set of 30 media sources chosen according to their prominence and age. To be specific, in method (c) we applied the Louvain community detection algorithm [48] to the empirical network M_{ss} which measures the relation between media-sources according to the number of times individuals were paired in articles from a given media source. To obtain this network we first calculated the weighted bi-partite network between individuals and media sources by tallying the number of articles associated with individual i by media source s, yielding the matrix M_{si}. We then projected this bi-partite network onto the space of media sources using the matrix transformation $M_{ss} = M_{si}M_{si}^T$, where the superscript T denotes the matrix transpose; we then set the diagonal elements $M_{ss} = 0$ since we are not concerned with the self-association. This straightforward bipartite projection method produces an intuitive measure of association for any two media sources derived from the number of articles from source s and s' who feature the same individual i. It is

worth pointing out that the Louvain community detection algorithm is unsupervised and involves just a single resolution parameter, and thus yields an exogenous classification of the s.

In summary, these three counting methods provide complementary perspectives on the visibility of CCC and CCS in the media. For each method we count the number of distinct media articles featuring CCC and/or CCS. Supplementary Figure 5 shows the comparison of group tallies for all permutations of CC group size and Media Source groups defined according to these three counting methods. The results are consistent and robust – indicating that CCC have nearly equal or significantly greater media visibility in all permutations with the exception for prominent media sources when only published scientists are considered – ie. Supplementary Figure 5b when comparing the subset of 224 CCC and 224 CCS.
